# Challenging the paradigm: non-canonical exoprotease cheating in clinical *Pseudomonas aeruginosa* isolates

**DOI:** 10.1093/femsec/fiaf106

**Published:** 2025-10-22

**Authors:** Katya Dafne Guadarrama-Orozco, Diego Armando Esquivel-Hernández, Miguel Ángel Islas-Tolentino, Fohad Mabood Husain, Héctor Quezada, Selene García-Reyes, Bernardo Franco, Diana Laura Marroquin-Mendiola, María Guadalupe Lucero-Gil, Lorena Paola Olvera-Falfan, Ángel Yahir Estrada-Velasco, Misael Josafat Fabián del Olmo, Miguel Cocotl-Yañez, María Tomas, Betsy Anaid Peña-Ocaña, Toshinari Maeda, Altaf Khan, Mohammed Arshad, Rafael Cantón, Antonio Oliver, Timothy J Kidd, Alejandra Valdez, Frederic Cadet, Shotaro Toya, Nicolas Fontaine, Corina-Diana Ceapă, Joy Kirigo, Thomas K Wood, Rodolfo García-Contreras

**Affiliations:** Departamento de Microbiología y Parasitología, Facultad de Medicina, Universidad Nacional Autónoma de México, Mexico City 04510, Mexico; Departamento de Procesos y Tecnología, Universidad Autónoma Metropolitana Cuajimalpa, Mexico City 05348, Mexico; Departamento de Nefrología, Hospital de Especialidades Dr. Antonio Fraga Mouret Centro Médico Nacional La Raza, Mexico City 02990, Mexico; Department of Food Science and Nutrition, College of Food and Agriculture Sciences, King Saud University, Riyadh 11451, Saudi Arabia; Laboratorio de Investigación en Inmunología y Proteómica, Hospital Infantil de México Federico Gómez, Ciudad de México 06720, Mexico; Programa de Investigadores por México-Secretaría de Ciencia, Humanidades, Tecnología e Innovación, Ciudad de Mexico 03940, Mexico; Instituto de Química, UNAM, Ciudad de Mexico 04510, Mexico; Departamento de Biología, División de Ciencias Naturales y Exactas, Universidad de Guanajuato, Noria Alta S/N, Guanajuato 36050, Mexico; Departamento de Microbiología y Parasitología, Facultad de Medicina, Universidad Nacional Autónoma de México, Mexico City 04510, Mexico; Departamento de Microbiología y Parasitología, Facultad de Medicina, Universidad Nacional Autónoma de México, Mexico City 04510, Mexico; Departamento de Microbiología y Parasitología, Facultad de Medicina, Universidad Nacional Autónoma de México, Mexico City 04510, Mexico; Departamento de Microbiología y Parasitología, Facultad de Medicina, Universidad Nacional Autónoma de México, Mexico City 04510, Mexico; Departamento de Microbiología y Parasitología, Facultad de Medicina, Universidad Nacional Autónoma de México, Mexico City 04510, Mexico; Departamento de Microbiología y Parasitología, Facultad de Medicina, Universidad Nacional Autónoma de México, Mexico City 04510, Mexico; Multidisciplinary and Translational Microbiology group (MicroTM),, Biomedical Research Institute of A Coruña (INIBIC), Microbiology Service, University Hospital of A Coruña (CHUAC), University of A Coruña (UDC), A Coruña 15006, Spain; Departamento de Bioquímica, Instituto Nacional de Cardiología, Mexico City 14080, Mexico; Department of Biological Functions Engineering, Graduate School of Life Science and Systems Engineering, Kyushu Institute of Technology, 2-4 Hibikino, Wakamatsu-ku, Kitakyushu 808-0196, Japan; Department of Pharmacology, King Saud University, Central Laboratory, Riyadh 11451, Saudi Arabia; Dental Health Department, College of Applied Medical Sciences, King Saud University, Riyadh 11451, Saudi Arabia; Servicio de Microbiología. Hospital Universitario Ramón y Cajal and Instituto Ramón y Cajal de Investigación Sanitaria, CIBER de Enfermedades Infecciosas (CIBERINFEC), Instituto de Salud Carlos III, Madrid 28034, Spain; Microbiology, Hospital Son Espases, Instituto de Investigación Sanitaria Illes Balears (IdISBa), CIBERINFEC, Palma 07120, Spain; Pathology Queensland—Central Microbiology Laboratory, Queensland Health, Brisbane, Queensland 4029, Australia; PROIMI, CONICET (Planta Piloto de Procesos Industriales Microbiológicos), Tucuman 4000, Argentina; Departamento de Biología Molecular y Biotecnología, Instituto de Investigaciones Biomédicas, Universidad Nacional Autónoma de México, Apdo. Postal 70228, C. P. 04510, University City, CDMX, Mexico; Artificial Intelligence Department, PEACCEL, AI for Biologics, Paris 75013, France; Department of Biological Functions Engineering, Graduate School of Life Science and Systems Engineering, Kyushu Institute of Technology, 2-4 Hibikino, Wakamatsu-ku, Kitakyushu 808-0196, Japan; Artificial Intelligence Department, PEACCEL, AI for Biologics, Paris 75013, France; Instituto de Química, UNAM, Ciudad de Mexico 04510, Mexico; Department of Chemical Engineering, Pennsylvania State University, University Park, PA 16802-4400, United States; Department of Chemical Engineering, Pennsylvania State University, University Park, PA 16802-4400, United States; Departamento de Microbiología y Parasitología, Facultad de Medicina, Universidad Nacional Autónoma de México, Mexico City 04510, Mexico

**Keywords:** clinical strains, exoprotease, experimental evolution, LasR, quorum sensing, sociobiology

## Abstract

*Pseudomonas aeruginosa* is a model organism for studying social behaviors in bacteria, such as the exploitation of exoprotease by social cheaters. The current paradigm holds that continuous culture of exoprotease-producing individuals with protein as the sole carbon source selects for exoprotease non-producers mutants with an impaired quorum-sensing regulator, LasR, which controls exoprotease expression. However, recent studies reveal that some isolates lacking functional LasR still produce exoproteases under the control of another regulator, RhlR. Here, we extended this study to two clinical strains, AUS 411 and AUS 531, isolated from cystic fibrosis patients and harboring functional LasR. Surprisingly, in AUS 411, exoprotease-non-producers appeared from the first growth passage, but most cells lost exoprotease production only transiently, with stable non-producers isolated only in late passages. In contrast, AUS 531 slowly selected stable non-producers with limited cheating ability, which neither accumulated to high proportions nor caused population collapses. Contrary to the paradigm, these non-producers had no inactivating mutations in *lasR* yet were more fit than laboratory-derived *lasR* deletion mutants in both casein and casamino acid media. Our findings demonstrate that social behavior can differ significantly from that in reference strains, suggesting that some *P. aeruginosa* strains evolve quorum-sensing networks with robust resistance to exploitation.

## Teaser

Our research reveals how some cystic fibrosis-derived *Pseudomonas aeruginosa* strains resist social cheating, preventing population collapses despite exoprotease-non-producer mutants.

## Introduction

*Pseudomonas aeruginosa*, an opportunistic bacterial pathogen, possesses numerous virulence factors, including the public goods siderophores and exoproteases, enabling host colonization and infection establishment (Rutherford and Bassler 2012, Garcia-Reyes et al. 2021). The expression of exoproteases is regulated by a well-characterized communication system called quorum sensing (QS). Three QS systems are described in *P. aeruginosa*, based on the production and detection of signaling molecules known as autoinducers (AIs). Two systems, Las and Rhl, utilize the acyl-homoserine lactones (HSLs)—C12-HSL and C4-HSL, respectively—as signaling molecules. A third system, PQS, relies on quinolones as AIs. When AIs reach a threshold concentration, they bind to their respective receptors—LasR, RhlR, or PqsR—forming AI-receptor complexes that activate virulence gene expression as well as genes for metabolic traits. For instance, LasR primarily regulates genes related to the production of exoproteases, elastases, and hydrogen cyanide, as well as the catabolism of adenosine (Rutherford and Bassler 2012, Garcia-Reyes et al. 2021), while RhlR controls genes involved in pyocyanin, rhamnolipids, hydrogen cyanide, siderophores, elastases, and alkaline protease production (Papenfort and Bassler 2016, Garcia-Reyes et al. 2021). The PQS system influences genes linked to biofilms, proteases, elastases, pyocyanin, and rhamnolipids (Garcia-Reyes et al. 2021, Chadha et al. 2022).

The three QS systems—Las, Rhl, and Pqs—are hierarchically interconnected. In nutrient-rich environments, the Las system initiates the signaling cascade (Smith and Iglewski 2003, Kostylev et al. 2019), while in low-phosphate conditions, Rhl takes the lead, rendering Las dispensable (Soto-Aceves et al. 2021). The Las system regulates target genes, including Rhl and Pqs (Gambello and Iglewski 1991). The Rhl system represses Pqs activity, while PqsE encoded in the *pqsABCDE* operon (involved in PQS synthesis) enhances RhlR function, completing a complex regulatory loop (Garcia-Reyes et al. 2020, Garcia-Reyes et al. 2021, Garcia-Reyes et al. 2021, Borgert et al. 2022).

Public goods are resources or molecules produced by individual bacterial cells that are secreted into the extracellular environment and can be used by other cells in the population, regardless of whether those cells contributed to their production. These goods are typically costly to produce but provide a collective benefit to the bacterial community. *Pseudomonas aeruginosa* produces siderophores that are small, iron-chelating molecules secreted by bacteria to scavenge iron from the environment and exoproteases that are enzymes secreted by bacteria that break down proteins in the environment into smaller peptides or amino acids, which can then be taken up as nutrients, and both are public goods since the benefits associated with their production, such as iron acquisition or carbon, nitrogen, and energy sources, are available for the whole bacterial population. This population includes not only those cooperative individuals that produce the public goods, but also non-producing cells. Hence, these traits are susceptible to cheating; accordingly, studies have shown that mutants unable to produce exoproteases or siderophores can be selected in competition with producers, demonstrating these virulence factors are susceptible to exploitation (Diggle et al. 2007, Sandoz et al. 2007, Harrison et al. 2017, Loarca et al. 2019, Tostado-Islas et al. 2021). In experimental evolution with protein as the sole carbon source, spontaneous exoprotease-non-producers emerge and are rapidly selected. Excessive accumulation of these non-producers can halt culture growth due to overexploitation of cooperators, leading to a population collapse (tragedy of the commons) (Sandoz et al. 2007, Dandekar et al. 2012, Loarca et al. 2019). Environmental factors—such as medium viscosity (Kummerli et al. 2009), nitrogen source variation (Lai et al. 2018), additional carbon sources (Dandekar et al. 2012), and stressors like reactive oxygen species (Garcia-Contreras et al. 2015) or bacteriophages (Saucedo-Mora et al. 2017, Huelgas-Mendez et al. 2023) influence exploitation outcomes, favoring either cheaters or cooperators.

Most studies on exoprotease exploitation by social cheaters have focused on reference strains PAO1 and PA14, with limited data on clinical or environmental isolates. In 2019, we demonstrated that a clinical strain from a burn patient exhibited behavior similar to reference strains (Loarca et al. 2019). Later, in studies with an environmental isolate (Huelgas-Mendez et al. 2023) and a cystic fibrosis isolate (Chen et al. 2019), RhlR controlled exoprotease expression rather than LasR. During continuous growth with casein as the sole carbon source, exoprotease-non-producers were selected, resulting in rapid population collapses due to overexploitation. Notably, these naturally selected non-producers were not *rhlR* mutants but had defects in quinolone signaling (Chen et al. 2019) or reduced *rhlR* expression (Huelgas-Mendez et al. 2023), the latter complemented by extra *rhlR* copies. Prophage activation in ID4365 accelerated collapses (Huelgas-Mendez et al. 2023). In contrast, a recent study found that in three of four cystic fibrosis *lasR*-null isolates, *rhlR*-null cheaters emerged during serial growth in casein, while in one isolate, *rhlR* mutants were outcompeted by the wild-type (Cruz et al. 2025).

Here, we investigated two clinical isolates from cystic fibrosis patients of the epidemic clone ST274-CC274 (Ambroa et al. 2020), both of which exhibited functional LasR through experimental evolution in media containing protein as the sole carbon source, as well as genomic sequencing, transcriptomics, proteomics, and phenotypic analysis characterization. We found mild exoprotease exploitation; however, it was not a result of *lasR* mutant selection during continuous growth in casein, challenging the canonical paradigm observed in reference strains.

## Material and methods

### Strains and growth conditions

The reference strain PAO1 and its isogenic *lasR* mutant (*lasR* gene interrupted with apramycin cassette) were obtained from the collection of Prof. Gloria Soberón Chávez at the Biomedical Research Institute of UNAM. Clinical strains from cystic fibrosis patients, belonging to the international CF clone ST274-CC274, were previously described (Ambroa et al. 2020). All other colonies derived from the evolution experiment and the AUS 531 *lasR* insertion (apramycin cassette) mutant were generated in this study (see Supplementary Material for the methodology employed to construct the mutant and Supplementary Table S4 for the description of the *P. aeruginosa* strains). At least three independent precultures per experiment were grown in LB medium or LB medium supplemented with 100 µg/mL gentamicin for the AUS 531 *lasR* mutant at 37°C with shaking at 200 r/m for 16–18 h. For the evolution experiments, serial growth passages were conducted in M9 minimal medium under the same conditions as the precultures, supplemented with 0.25% caseinate as the sole carbon source. The first serial cultures were initiated by inoculating the caseinate medium with LB precultures to an initial turbidity of Abs 600 nm ≈ 0.05. Subsequent serial cultures were inoculated using the previous caseinate culture, adjusted to an initial turbidity of Abs 600 nm ≈ 0.05.

### Activity of exoprotease and quorum-sensing signals

Exoprotease activity was measured by recording the caseinolytic activity of strain supernatants grown in LB using the azocasein assay, as reported in Loarca et al. (2019). HSLs were quantified via bioassays using two biosensor strains: *Chromobacterium violaceum* CV026 for C4-HSL and *Pseudomonas putida* F117 (pKR-C12) for C12-HSL detection, as described previously in Torres et al. (2024). HSLs were determined using two independent colonies *per* strain. PQS levels were determined using the biosensor PAOpqsA:: lux (Fletcher et al. 2018). Briefly, overnight precultures were diluted to an OD600 of 0.01 and incubated at 37°C in 25 mL of LB for 24 h with shaking. Cultures were then centrifuged at 5000 r/m for 10 min at 4°C, and supernatants were filtered through 0.22-μm pore filters. Subsequently, 75 μL of supernatant was mixed with 75 μL of biosensor, loaded into a Corning black flat-bottom plate, and incubated for 8 h at 37°C. Luminescence and growth were measured using a Synergy H1 microplate reader (BioTek), with luminescence expressed as relative units per optical density at 600 nm (OD_600_). For PQS determination, three independent colonies *per* strain were used.

### DNA extraction and genomic analysis

Genomic DNA was extracted from overnight cultures using the DNeasy UltraClean Microbial Kit (Qiagen Inc., Valencia, CA, USA). The DNA concentration was determined with a NanoDrop spectrophotometer. Genome sequencing was performed using Nanopore technology by Plasmidsaurus (San Francisco, CA, USA). This involved constructing an amplification-free library using v14 chemistry and sequencing with a primer-free protocol on R10.4.1 flow cells and Oxford nanopore sequencing technology. The assembly pipeline used by Plasmidsaurus was as follows: Remove the bottom 5% of the worst FastQ reads via Filtlong v0.2.1 (default parameters). Downsample the reads to 250 Mb via Filtlong to create a rough sketch of the assembly with Miniasm v0.3. Using information acquired from the Miniasm assembly, re-downsample the reads to ∼100x coverage (do nothing if there is not at least 100x coverage) with a heavy weight applied to removing low-quality reads (helps small plasmids stick around). Run a Flye v2.9.1 assembly with parameters selected for high-quality ONT reads. Polish Flye assembly via Medaka v1.8.0 using the reads generated in step 3, run several analyses: annotation was conducted with Bakta v1.6.1 (Schwengers et al. 2021), contig analysis Bandage v0.8.1 genome completeness and contamination CheckM v1.2.2 species/plasmid identification Mash v2.3 against RefSeq genomes+plasmids, Sourmash v4.6.1 against GenBank CheckM v1.2.2. Genome visualization and analysis were conducted using Qiagen CLC Genomics Workbench 25. Sequence identity was assessed using several tools: Average Nucleotide Identity (ANI) analysis with the Proksee (Grant et al. 2023) tool for prokaryotic genome maps and FastANI (Jain et al. 2018), as well as sequence identity mapping in Qiagen CLC Genomics Workbench. Genes were annotated using Bakta (Schwengers et al. 2021).

For the phylogenetic tree, the genomes were annotated using RAST tool kit (RASTtk) in the BV-BRC platform (Olson et al. 2023). Mutation analysis was performed using the variation analysis pipeline BWA-mem-strict with the FreeBayes single-nucleotide polymorphism (SNP) caller. ProgressiveMauve (Darling et al. 2010) was used for whole-genome alignment. The tree was generated by implementing RAxML (Stamatakis 2014), permitting one gene deletion and one duplication per genome. The tree represents the alignment of 1000 single-copy shared genes, using MAFFT for alignment.

### Sequencing of *lasR* gene

For the sequencing of *lasR* genes of selected AUS 531 non-exoprotease producers, genomic DNA was extracted as detailed before, and PCR was done using the primers *lasR* fw 5′- AGGAAGCCGGGATTCTCGGA- 3′ and *lasR* Rv 5′-GAGAATGGCGAGAACCTGCC-3′ that amplify a fragment of 920 bp. PCR products were then purified using the NucleoSpin Gel and PCR Clean-up kit (Macherey-Nagel, Duren, Germany) and sequenced by the Sanger method in both forward and reverse senses at Quintara Bioscience (https://www.quintarabio.com/). The resulting sequences were aligned and analyzed using the Mega 12 software (https://www.megasoftware.net/).

### Transcriptomics

For transcriptomic analysis, bacteria (two independent colonies *per* strain) were grown to the exponential phase in LB. One-milliliter aliquots were centrifuged at 13 000 r/m for 20 s and flash-frozen in a dry ice-ethanol bath to prevent mRNA degradation (Richmond et al. 1999). Frozen samples were stored at −80°C until use. Bacterial pellets were disrupted using a bead beater, and RNA was extracted using the RNeasy Kit (Qiagen Inc., Valencia, CA, USA) according to the manufacturer’s instructions (Shekhar and Maeda 2022). RNA integrity was verified by electrophoresis on a 1.4% agarose gel. RNA was reverse transcribed using the PrimeScript RT Reagent Kit Perfect Real Time (TAKARA Bio Inc., Japan) as described in Shekhar and Maeda (2022) and sequenced with an Illumina MiSeq system. Genomic DNA libraries were prepared with the Nextera XT DNA Library Preparation Kit (Illumina, San Diego, CA, USA) and sequenced to generate 300-bp paired-end reads. Read quality was assessed with FastQC v1.0.1, and low-quality reads and adapters were removed using Trimmomatic v0.36. Genome assembly was performed using SPAdes software on the KBase Predictive Biology platform v3.15.3 (Bankevich et al. 2012, Arkin et al. 2018).

### Bioinformatics analysis for RNA-seq data

Analyses were conducted using the “Reference-based RNA-Seq Data Analysis” pipeline (https://training.galaxyproject.org/training-material/topics/transcriptomics/tutorials/ref-based/tutorial.html) on the Galaxy server, with minor modifications. RNA-seq reads from *P. aeruginosa* cultures were analyzed using FastQC (Wingett and Andrews 2018) and trimmed with Cutadapt (Kechin et al. 2017) with a minimum quality cutoff (Q) of 20. High-quality reads were mapped with RNA-STAR (Dobin et al. 2013) to the complete reference genome of P*. aeruginosa* PAO1 (GCF_000006765.1) (Stover et al. 2000), obtained from the Pseudomonas Genome Database (https://www.pseudomonas.com/strain/download). Default RNA-STAR parameters for prokaryotes were used, with CDS as the genetic element for splice junctions. Read counts per gene were determined using FeatureCounts (Liao et al. 2014) and RNA-STAR results, normalized with the DESeq2 rlog function. Differential gene expression analysis was performed with DESeq2, comparing strain AUS 531 34.7 (case) and strain AUS 531 (control).

### Data accessibility

Transcriptomics data are available on the NCBI platform under accession number PRJEB86431. Proteomic datasets are stored in the MassIVE repository (https://massive.ucsd.edu/ProteoSAFe/static/massive.jsp) under project accession PXD061353 (DOI: 10.6019/PXD061353). Genome data are available on the NCBI under BioProject: PRJNA1245488, Sequence Read Archive (SRA): SRR32970825, Accession Number: CP187262, and Biosample ID: SAMN47757631 for AUS 531 and Biosample ID SAMN47757632 for AUS 531 24.7.

### Construction of *lasR* mutant in AUS 531

The *P. aeruginosa* AUS 531 *lasR* mutant was generated by homologous recombination using a plasmid-borne insertion-deletion, as described in Choi and Schweizer (2005). The plasmid pEX-*lasR*::Apra was constructed as follows: a 1896-bp fragment containing the *lasR* upstream region, gene, and downstream region was PCR-amplified from AUS 531 genomic DNA using oligonucleotides UplasRHd and DwlasRHd. The PCR product was cloned into the pMINIT2.0 vector (New England Biolabs, Ipswich, MA, USA) to form pMINIT2.0-UplasRDw. Reverse PCR using pMINIT2.0-UplasRDw as a template with oligonucleotides 5lasRKpnI and 3lasRKpnI removed the 760-bp *lasR* gene, yielding pMINIT2.0-UpDw. The apramycin resistance gene (*aprA*, 1370 bp) was amplified from plasmid pIJ773 using oligonucleotides ApraFW and ApraRV. Both pMINIT2.0-UpDw and the *aprA* PCR product were digested with DpnI, phosphorylated, and ligated using the Q5® Site-Directed Mutagenesis Kit (New England Biolabs, catalog no. E0554S) to generate pMINIT2.0-UpApraDw. This plasmid was digested with HindIII, and the resulting 2500-bp fragment was cloned into the HindIII sites of the suicide vector pEX18Amp, yielding pEX-*lasR*::Apra. Mutants were selected on LB-agar plates containing 100 µg/mL gentamicin (Gm), as the *aprA* gene also confers resistance to Gm.

### Proteomic analysis

The exoproteome of AUS 531 and its derived non-exoprotease producer 34.7 was analyzed from three independent LB cultures per strain, grown at 37°C with shaking for 16 h. Supernatants were filtered (0.22-μm filters) and subjected to two protein precipitation steps. First, 10 mL of filtered supernatant were mixed with 40 mL of cold acetone and incubated overnight at −20°C. After centrifugation (20 000 × g, 5 min, 4°C), the pellet was washed with 300 µL of cold acetone, centrifuged, and air-dried. Second, the pellet was resuspended in 300 µL methanol, 100 µL chloroform, and 300 µL water, vortexed for 10 s, and centrifuged (20 000 × g, 15 min, 4°C). The interphase was washed with 450 µL methanol, centrifuged again (20 000 × g, 5 min, 4°C), dried, and resuspended in 100 µL of 1.2 M urea, 50 mM ammonium bicarbonate, and 0.02% ProteaseMAX (Promega, V2071, Madison, WI, USA). Protein concentration was determined with the QuantiPro BCA Assay Kit (Merck Sigma-Aldrich, St. Louis, MO, USA). Fifty micrograms of protein were reduced with 10 mM dithiothreitol (DTT) at 56°C for 20 min, alkylated with 20 mM iodoacetamide in the dark for 20 min at room temperature, and digested with 3 µg trypsin (Promega, V5280, Madison, WI, USA) in 50 mM ammonium bicarbonate for 18 h at 37°C. Formic acid was added to 0.5%, and peptides were desalted (Waters Sep-Pak cartridges, Milford, MA, USA) and vacuum-dried.

Chromatographic separation and mass spectrometry were performed as described in Loarca et al. (2019). Briefly, dried peptides were reconstituted in 20 µL of 5% acetonitrile and 0.1% formic acid, and 1 µg was injected into a Dionex Ultimate 3000 system (Thermo Fisher Scientific, Waltham, MA, USA). Peptides were separated on an Acclaim PepMap C18 column (75 µm × 50 cm, Thermo Fisher Scientific) at 300 nL/min with a linear gradient (2%–40% Buffer B over 86 min; Buffer A: 0.1% formic acid in water; Buffer B: 0.1% formic acid in acetonitrile). Electrospray ionization was performed using a CaptiveSpray source (Bruker, Billerica, MA, USA) with nitrogen assistance (0.2 bar), and mass spectra were acquired with a quadrupole time-of-flight mass spectrometer (Impact II, Bruker). Raw files were analyzed with MaxQuant (v2.1.3.0) using Label-Free Quantification (LFQ) and the *P. aeruginosa* PAO1 reference proteome (UniProt UP000002438, accessed January 2025). Valid identifications required at least two unique peptides in two biological replicates. Differences in abundance were assessed with Perseus (v1.6.15.0) using a two-sample test of log2-transformed LFQ intensities (permutation-based FDR, Student’s *t*-test, FDR = 1%, fold change > 1.5). Fold changes were calculated from median untransformed LFQ intensities.

### Bacterial competitions

Competitions were conducted in M9 minimal medium supplemented with casein or casamino acids, as described in Tostado-Islas et al. (2021). Cocultures were initiated with approximately 5 × 10^7 bacteria/mL, with initial proportions of cooperators and cheaters estimated from the preculture growth. Initial and final strain percentages were determined based on exoprotease production (PAO1, AUS 531) or absence (PAO1 *lasR*, AUS 531 34.7) or gentamicin resistance (AUS 531 *lasR*). All competitions were performed using three independent colonies *per* strain.

### Statistical analysis

Data were analyzed using the IBM SPSS Statistics software, and the Student’s *t*-test was applied. Differences were considered significant when *P* values were lower than 0.05.

## Results

### Exoprotease production in AUS 411 and AUS 531 is variable and controlled by homoserine lactone autoinducers

Before the evolution experiments, exoprotease production (caseinolytic activity) was assessed in multiple colonies of AUS 411 and AUS 531 (ST274-CC274 clones (Ambroa et al. 2020)). Both strains exhibited high variability in exoprotease activity, unlike reference strain PAO1 and burn patient isolate P729, which had less variability in their exoprotease production (Supplementary Fig. S1); similar outcomes in experimental evolution were seen during growth in casein as the sole carbon source (Loarca et al. 2019). Production of long-chain (C12-HSL, Supplementary Fig. S2A) and short-chain (C4-HSL, Supplementary Fig. S2B) HSLs and quinolone signals (Supplementary Fig. S2C) was confirmed in both strains. Exoprotease activity was controlled by these HSL signals, as cleavage with AiiM HSL lactonase (Wang et al. 2010, Lopez-Jacome et al. 2019) inhibited caseinolytic activity (Supplementary Fig. S3).

### Serial growth in casein as the sole carbon source is stable in AUS 411 and AUS 531 and resistant to population collapses

**Figure 1.**
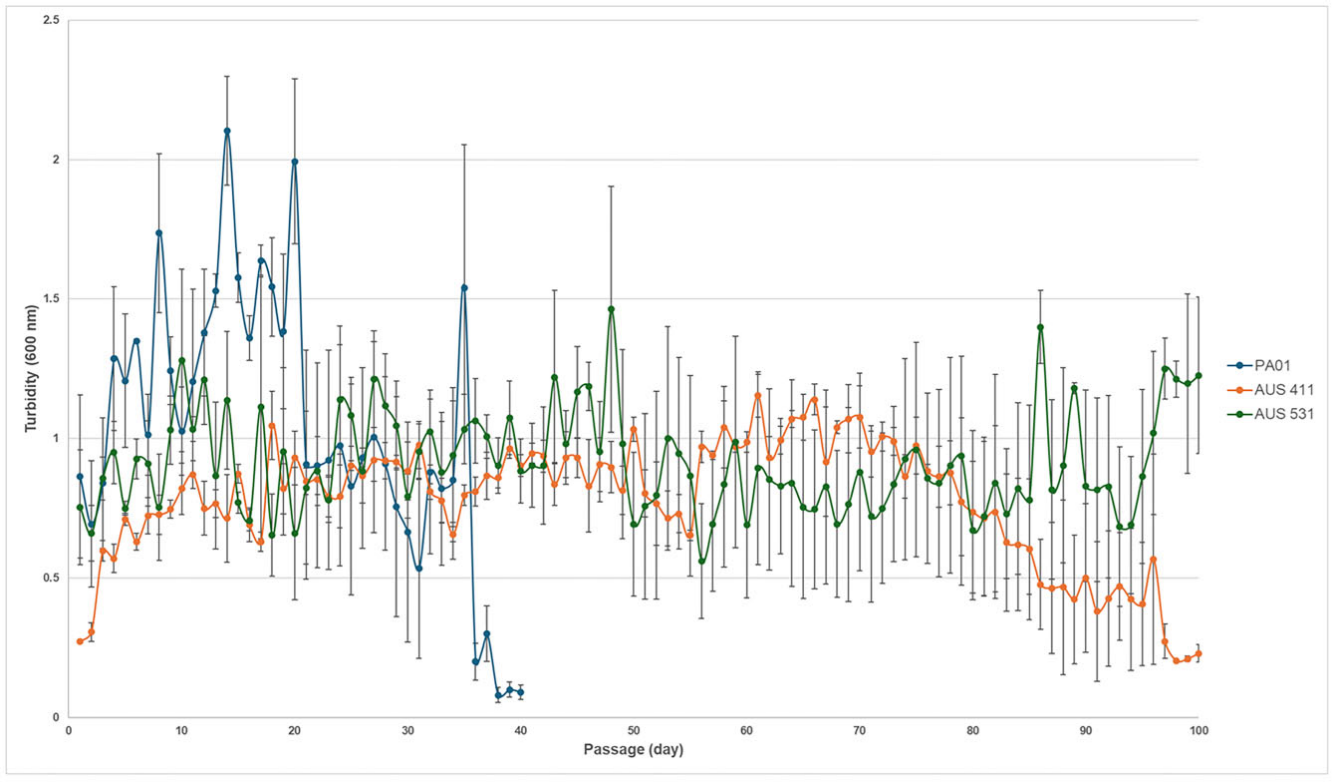
Serial growth of the PAO1, AUS 411, and AUS 531 strains in minimal medium with casein as the sole carbon source. Growth after 24 h of culture passage is shown; the experiments were performed using three independent cultures per strain, and the mean values ± SEM are shown.

**Figure 2.**
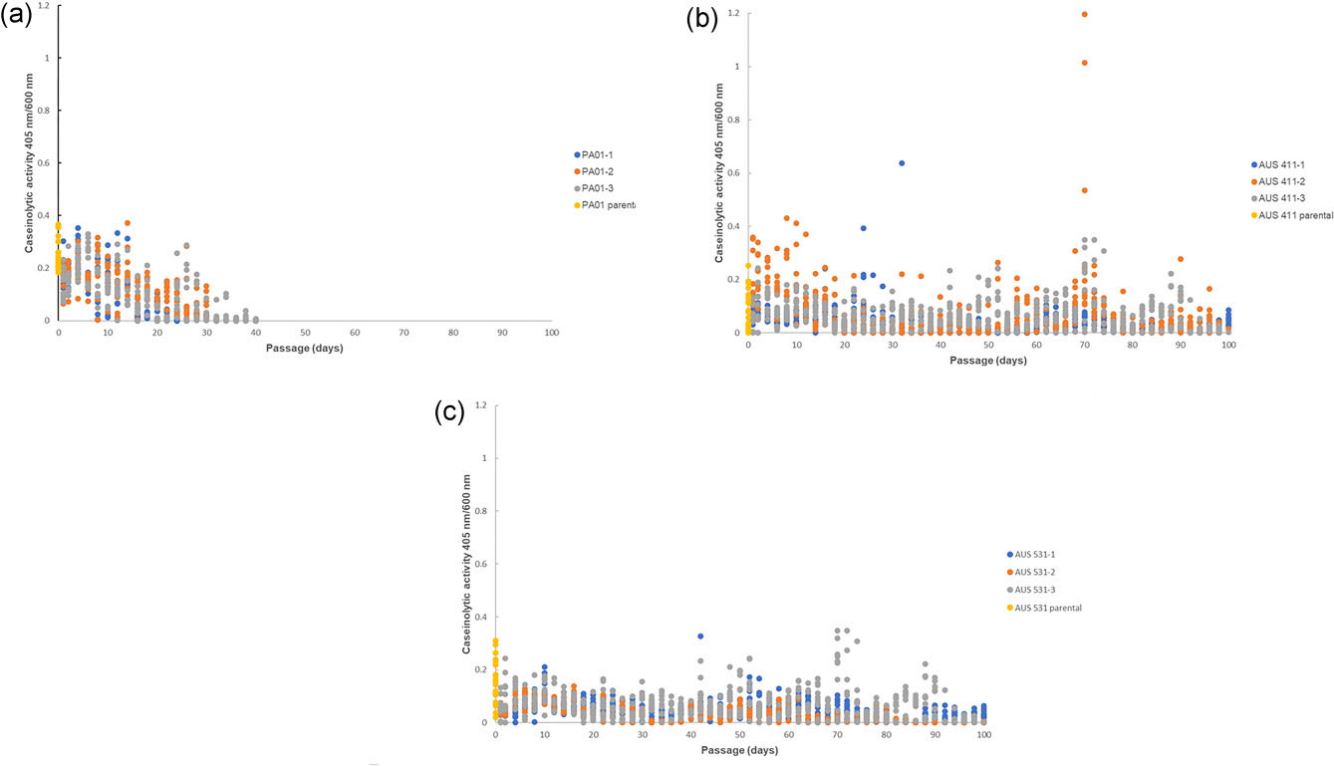
The caseinolytic activity of colonies isolated from the initial serial passage and consequent odd passages of the experiments in Fig. 1. Each dot represents the caseinolytic activity of an individual colony. The parental caseinolytic activity for each strain is shown for comparison (for 20 colonies cultured on LB). Panel a corresponds to PA01, panel b to AUS 411, and panel c to AUS 531.

To investigate the selection dynamics of exoprotease-non-producers and public goods exploitation, evolution experiments involving 100 serial passages in casein as the sole carbon source were performed. AUS 411 and AUS 531 (Supplementary Fig. S4), were selected for experimental evolution due to their high variability in exoprotease production (Supplementary Fig. S1), and PAO1, an archetypical clade 1 strain (Quiroz-Morales et al. 2022), was used for comparison. As expected, the maximal growth of PAO1 in casein medium decreased after 20 passages (Fig. 1), with collapses occurring between passages 24 and 38 (Supplementary Fig. S5A). Non-exoprotease producers (with very low or no detectable caseinolytic activity) emerged after passage 6 and accumulated progressively (Fig. 2A), consistent with PA14 (data not shown). In contrast, AUS 411 and AUS 531 exhibited stable growth, with no decrease until passage 74 for AUS 411, while two of three AUS 531 cultures showed no decline over 100 passages (∼400 generations). Although growth decreased in late passages for all AUS 411 cultures and one AUS 531 culture, no collapses occurred (Fig. 1 and Supplementary Figs S51B and S5C).

### Lack of selection of *lasR* mutants in clinical isolates during evolution in casein medium

**Figure 3.**
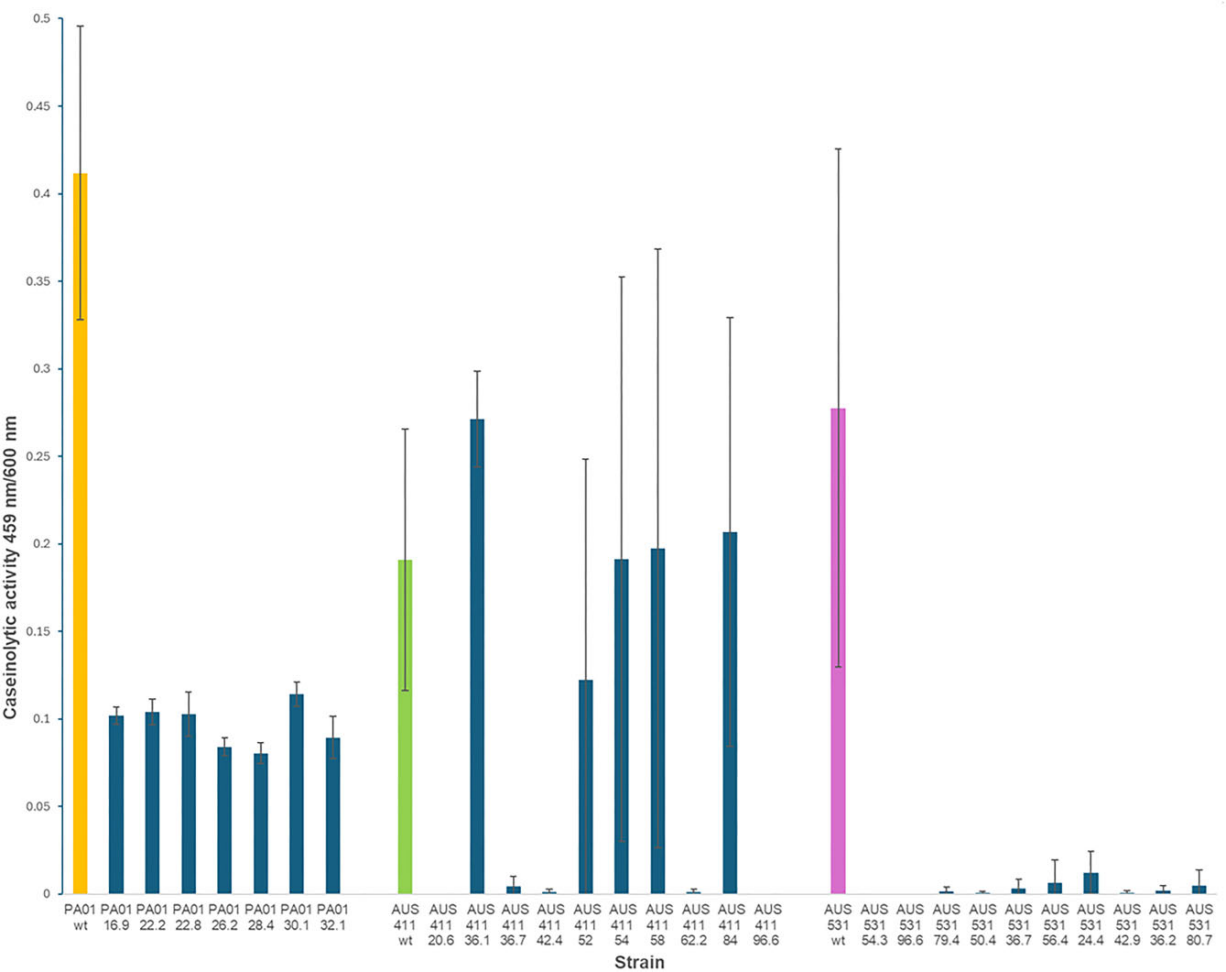
Caseinolytic activity from individual colonies that were isolated as non-exoprotease producers in the experiments in Fig. 1. Each colony was propagated, and the caseinolytic activity of 3 or 4 colonies derived from the original one was determined. from a non-exoprotease producer or the wild-type strain, the solid bar represents the mean, and the error bars represent the standard deviation of the caseinolytic activity for each non-exoprotease producer or wild-type strain.

Unlike PAO1, AUS 411 generated non-exoprotease producers from early passages, but their accumulation was not sustained in later passages (Fig. 2b). In AUS 531, non-producers also appeared early and showed weak accumulation only after passage 60 (Fig. 2c), differing from reference strains (Sandoz et al. 2007, Loarca et al. 2019, Tostado-Islas et al. 2021). Several apparent non-producers from AUS 411 and AUS 531 were isolated, cryopreserved, and compared with PAO1-derived non-producers. All PAO1 non-producers consistently generated low exoprotease producers’ colonies upon restreaking (Fig. 3). In contrast, many AUS 411 non-producers generated both caseinolytic-active and inactive colonies, indicating an unstable phenotype (Fig. 3). All AUS 531 non-producers consistently produced non-producing colonies, suggesting potential social cheaters (Fig. 3). AUS 531 was selected for further study, and two derived non-producers were characterized.

**Figure 4.**
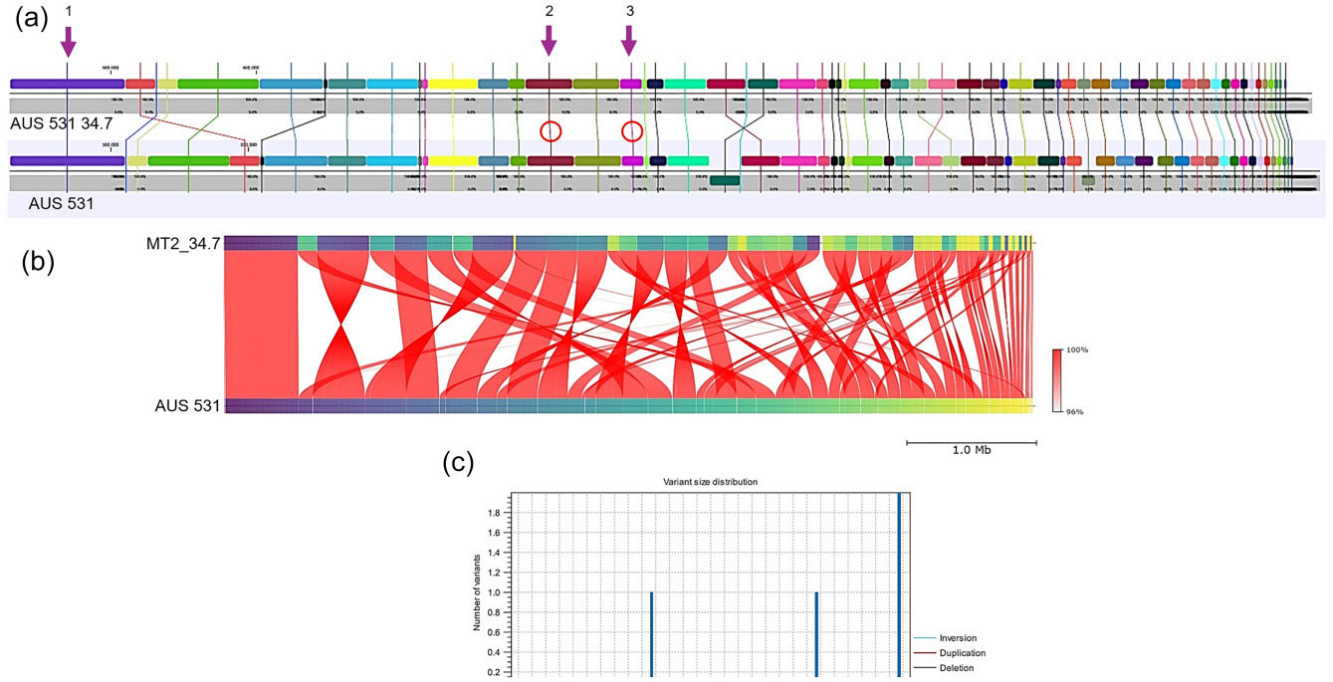
Genomic map comparison between parental AUS 531 strain with derivative AUS 531 34.7 strain. (a) Genomic maps showing the position of the assembled contigs, lines indicate the position of the homologous region in the AUS 531 parental strain. The purple arrows indicate the position of three insertions found in the whole-genome comparison; since the insertions are small, in the map, only the regions comprising these insertions are marked with a red circle. Visualization and analysis were carried out in Qiagen CLC Genomics Workbench 25. To assess the identity between the two sequences, several tools were used. (b) Average Nucleotide Identity analysis was conducted in the Proksee map of the AUS 531 34.7 genome map using FastANI and showed a 99.994 ANI index, with the homology distribution shown in a and other regions showing homology. To confirm the data shown, the lower graph presents a two-genome comparison (sequence identity mapping) conducted in Qiagen CLC Genomics Workbench, which displays the same pattern as shown in a. (c) A global estimate of variants present in the AUS 531 34.7 genome in comparison with the AUS 531 parental strain, showing three insertions present in the AUS 531 34.7 strain.

**Figure 5.**
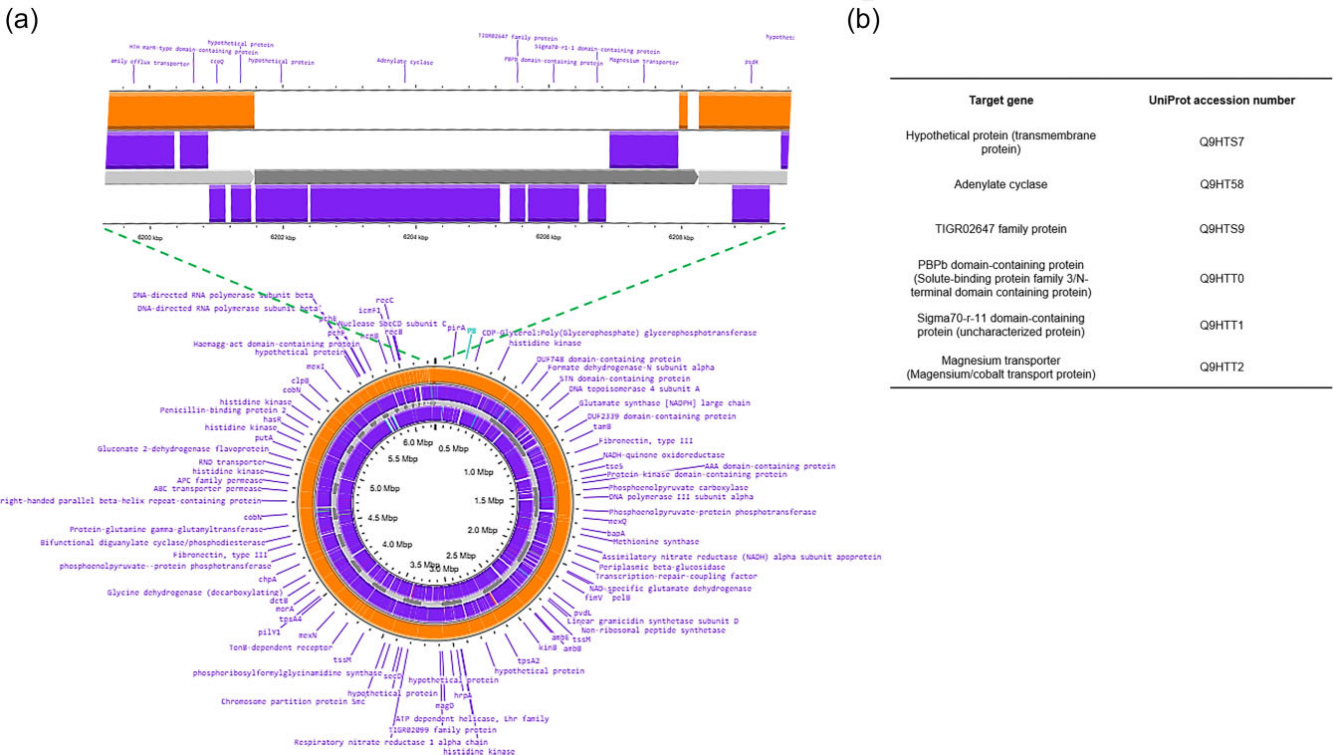
Genomic map showing the conservation between the AUS 531 parental strain and the AUS 531 34.7 derived strain. (a) The inner circle, the annotated genes are shown. In orange, the full genome alignment between the AUS 531 34.7 strain and the parental AUS 531 strain (orange circle). The largest insertion is shown in the zoomed-in region of the genome. (b) shows the UniProt accessions of the genes found in the insertion region. In parentheses, the UniProt annotation is shown, which differs from the annotation by Bakta.

Since *lasR* inactivation is a common mechanism for non-producer selection in casein (Sandoz et al. 2007, Wilder et al. 2011, Dandekar et al. 2012, Loarca et al. 2019), we hypothesized that AUS 531-derived non-producers were *lasR* mutants. Whole-genome sequencing of AUS 531 34.7, performed via Nanopore sequencing technology, revealed structural differences due to recombination (Fig. 4a). However, overall sequence identity and homologous section distribution were highly conserved (Fig. 4b). Strain 34.7 contained two small insertions (304 and 2435 bp) in non-coding regions and a 6889-bp insertion with six genes, including uncharacterized proteins, an adenylate cyclase, and a magnesium/cobalt transporter (Fig. 5a, b). Only 14 SNPs were identified, with two non-synonymous mutations in a peptidase and the RNA polymerase alpha subunit (Supplementary Table S1). Surprisingly, no mutations were found in the coding or promoter regions of *lasR, rhlR, lasB, lasA*, or other known QS or protease genes, indicating a non-canonical regulation of exoprotease loss in AUS 531 cheaters. Moreover, whole genome sequencing of the other non-exoprotease producer, AUS 531 24.7 via Nanopore, revealed similar features to those found in AUS 531 24.7, including an intact *lasR* gene (data not shown). In addition, the *lasR* gene of 5 additional non-exoprotease producers (20.2, 24.9, 26.3, 28.2, and 66.2) was sequenced by the Sanger method and found also no mutations in the coding region of *lasR*, further suggesting that in this strain, exoprotease non-producers selected during continuous growth in casein are not the result *lasR* mutations.

To complement the *lasR* sequencing results of the additional non-exoprotease producers, we found that they were unable to grow in casein as a sole carbon source (data not shown). In addition, their production of short and long HSLs signals was determined, and we found only one isolate (20.2) was able to produce short HSLs, and two isolates, 26.3 and 28.2, produced long HSLs (Supplementary Fig. S6).

### Stable non-exoprotease producers derived from AUS 531 are exoprotease cheaters

**Figure 6.**
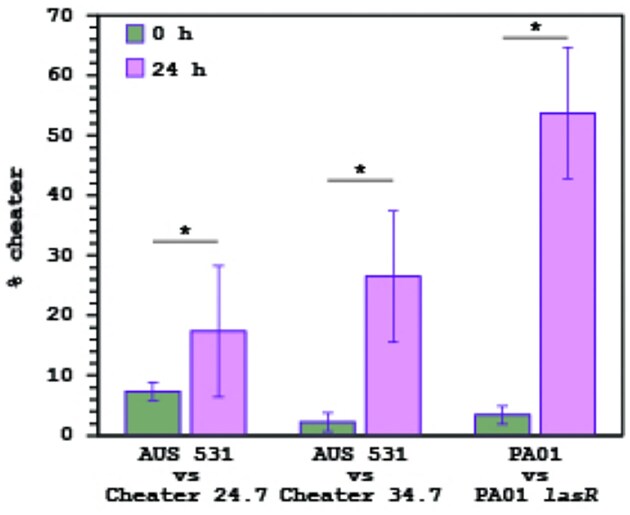
Competitions of AUS 531 and the derived non-exoprotease producers 24.7 and 34.7. The initial percentage of the non-exoprotease producers at the beginning of the experiments and after 24 h are shown, for comparison of the competence between PAO1, and its isogenic *lasR* mutant is shown. Competitions were performed in triplicate, and the average of the initial and 24 h percentages of non-exoprotease producers ± SEM are shown. Asterisk indicates significant differences (*P* ˂ 0.05 in a two-tailed *t*-test).

Given no collapses or significant non-producer accumulation in AUS 531, the fitness of non-producers AUS 531 24.7 and 34.7 was assessed in casein competitions with the parental strain. Both increased in frequency, confirming cheating ability (Fig. 6), though their selection was milder than that of a PAO1 *lasR* mutant against PAO1 wild-type (Fig. 6). In addition, to confirm AUS 531 24.7 and 34.7 were exoprotease cheaters, they were grown in caseinate as the sole carbon source, and as expected due their lack of exoprotease production, they showed a severe growth impairment after 20 h of cultivation; in contrast to the AUS 531 wild-type strain that showed robust growth (Supplementary Fig. S6).

**Figure 7.**
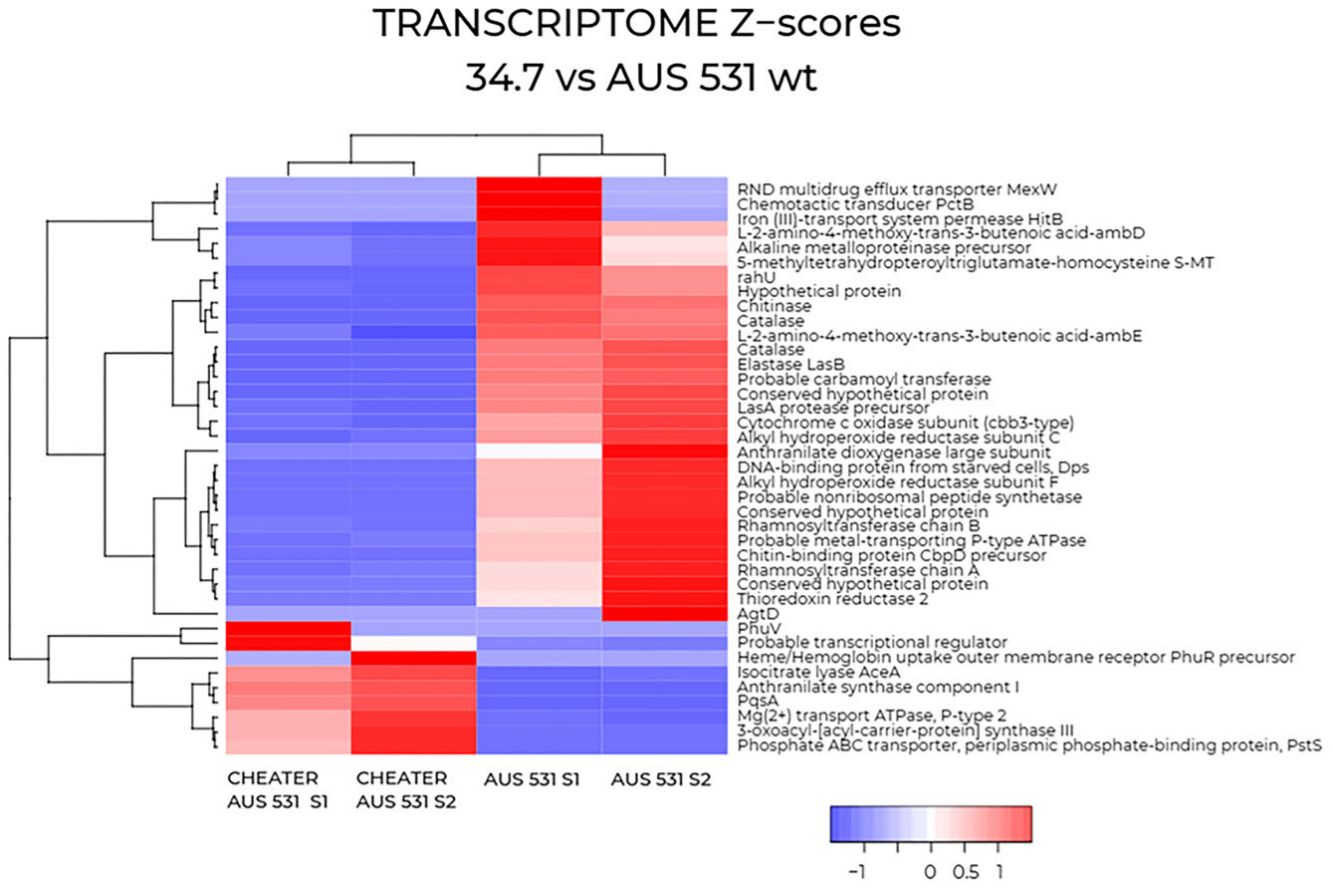
Transcriptomics. Heatmap indicating transcriptional changes between AUS 531 and its non-exoprotease producer 34.7; genes are clustered according to their functional category using the weighted pair group method with arithmetic mean. Two independent transcriptomes per strain are shown.

Proteomic and transcriptomic analyses were compared between AUS 531 34.7 and the wild-type. Proteomics of overnight culture supernatants showed LasA and LasB abundances were 365- and 72-fold higher in the wild-type, respectively, confirming these as the primary exoproteases impaired in 34.7 (Supplementary Table S2), consistent with reference strains (Cezairliyan and Ausubel 2017, Loarca et al. 2019). Other proteases more abundant in the wild-type included an immunomodulating metalloprotease (Q9I5W4) and Lon protease (Q9I2T9). Of 143 valid identifications, 85 showed significant abundance differences (18 more abundant and two exclusive to wild-type supernatants; Supplementary Table S2). Transcriptomics of late exponential-phase cultures revealed 32 genes upregulated and 8 downregulated in the wild-type (Fig. 7, Supplementary Table S3). Upregulated genes included QS-induced *lasA, lasB*, and *aprA*, as well as genes for rhamnolipid synthesis, chitinase, efflux pumps, and oxidative stress resistance. Downregulated genes in the wild-type (overexpressed in 34.7) included quinolone synthesis and phosphate/hemophore transporters. Notably, *lasR* and *rhlR* expression were unchanged.

### AUS 531 *lasR* mutant has a limited exoprotease exploitation ability and is less fit than naturally evolved AUS 34.7 in casein degradation products

The lack of *lasR* mutant selection in AUS 531 during casein passages contradicted the paradigm, potentially due to LasR controlling essential phenotypes or high residual exoprotease production in *lasR* mutants. However, an AUS 531 *lasR* interruption mutant was viable and showed robust growth in LB, with 60% of colonies producing no exoprotease and the rest moderate amounts, like PAO1 *lasR* mutants (data not shown). Thus, neither growth impairment nor high residual exoprotease activity explained the lack of selection of AUS 531 *lasR* mutants. Notably, AUS 531 *lasR* still produces both short and long HSL signals (Supplementary Fig. S6). However, despite AUS 531 *lasR* mutants showing low exoprotease production in the supernatants, they can still grow in casein as the sole carbon source, although to a lower extent than the parental strain (Supplementary Fig. S7), hence their ability to exploit exoprotease production of the parental strains may be limited by this factor.

**Figure 8.**
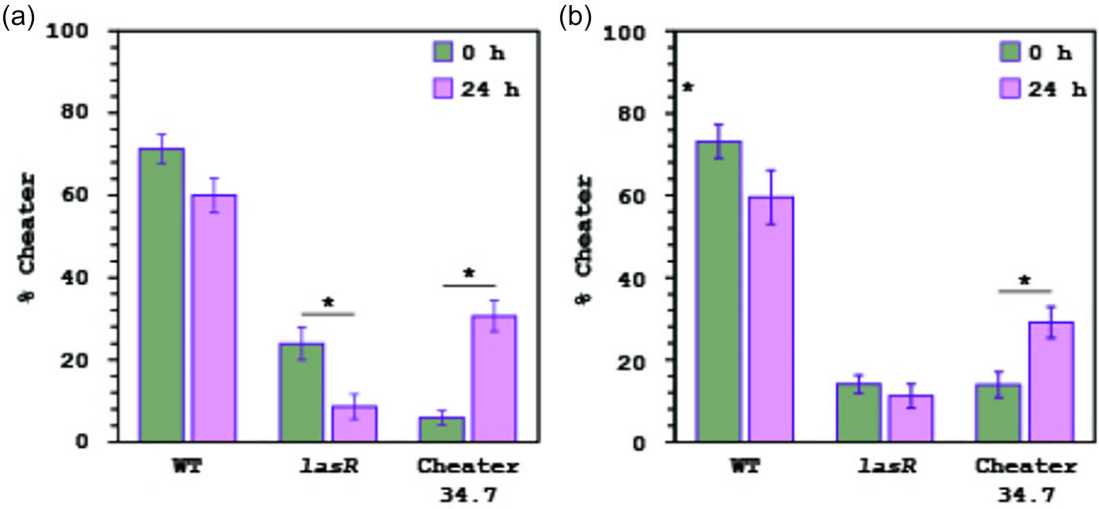
Competitions of AUS 531 and the non-exoprotease producer 34.7 and the isogenic AUS 531 *lasR* mutant in (a) casamino acids and (b) casein. The initial percentage of the 3 strains at the beginning of the experiments and after 24 h are shown. Competitions were performed in triplicate and the average of initial and 24 h percentages ± SEM are shown. Asterisk indicates significant differences (*P* ˂ 0.05 in a two-tailed *t*-test).

Alternatively, *lasR* mutants might poorly metabolize casein hydrolysis products. Growth rates in M9 with casamino acids showed the AUS 531 *lasR* mutant grew slower than the wild-type (1.23 ± 0.1 h^-1^ vs. 1.56 ± 0.14 h^-1^, *P* < 0.05, two-tailed *t*-test), unlike PAO1 *lasR* vs. wild-type (1.33 ± 0.14 h^-1^ vs. 1.16 ± 0.11 h^-1^). The 34.7 cheater’s growth rate (1.45 ± 0.22 h^-1^) matched the wild-type (Supplementary Fig. S8). Competitions in casamino acids confirmed higher selection of 34.7 over the *lasR* mutant against wild-type AUS 531 (Supplementary Fig. S9). While the tripartite competitions in casein and casamino acids showed 34.7 outcompeted the *lasR* mutant, increasing in frequency while the *lasR* mutant declined (Fig. 8), explaining the absence of classic *lasR* mutants in the evolution experiments.

## Discussion

The regulation of exoprotease production in *P. aeruginosa* through QS is a well-established paradigm, predominantly derived from studies of reference strains PAO1 and PA14, where LasR serves as the master regulator of exoprotease expression. Under this model, continuous growth with protein as sole carbon source selects for *lasR* mutants that cease exoprotease production, exploiting cooperators and often triggering population collapses—a phenomenon framed as a microbial “tragedy of the commons” (Sandoz et al. 2007, Dandekar et al. 2012, Loarca et al. 2019). However, emerging evidence, including from environmental and clinical isolates, suggests this paradigm is not universal. Some *lasR*-null strains sustain exoprotease production via alternative QS regulators like RhlR (Chen et al. 2019, Huelgas-Mendez et al. 2023, Cruz et al. 2025), revealing flexibility in QS hierarchies. Our study extends this complexity by demonstrating that two clinical isolates from cystic fibrosis (CF) patients, AUS 411 and AUS 531, both harboring functional LasR. These exhibit exoprotease exploitation dynamics that deviate markedly from those of reference strains. Notably, AUS 531 fails to select *lasR*-inactivated exoprotease-non-producers during prolonged casein growth, challenging the canonical view of QS-driven social cheating.

To our knowledge, this is the first report of a *P. aeruginosa* strain where exoprotease-non-producers emerge without *lasR* inactivation or reduced expression. In AUS 531, non-producers were selected, but their accumulation was weak, their cheating ability limited, and population collapses were absent, even though these strains harbor temperate prophages, which accelerated collapses in the *lasR*-null ID4565 strain via prophage activation (Huelgas-Mendez et al. 2023). This stability contrasts sharply with PAO1, where non-producers rapidly dominate and halt growth (Sandoz et al. 2007, Loarca et al. 2019). In AUS 411, non-producers appeared early but were transient, with stable cheaters emerging only in late passages, suggesting a dynamic interplay of regulatory or epigenetic factors. These findings underscore that those clinical isolates may possess QS networks with greater resilience to exploitation than laboratory-adapted strains, potentially reflecting adaptations to the complex, nutrient-variable environments of CF lungs.

The robustness of AUS 531′s cooperation network may stem from several factors. First, the absence of *lasR* mutations in non-producers, despite genomic changes (e.g. 304, 2435, and 6889-bp insertions), suggests that a non-canonical mechanism regulates exoprotease loss. “Notably, previous experimental evolution studies that selected for the simultaneous loss of exoprotease and pyoverdine production in PA14 revealed genomic rearrangements (deletions that included *lasR* and *lasI* genes) responsible for the loss of exoprotease production in exoprotease cheaters (Tostado-Islas et al. 2021). In contrast, in the present work, insertions, rather than deletions, may be responsible for the impairment of exoprotease production.”

Transcriptomic and proteomic analyses confirmed reduced LasA and LasB production in the AUS 531 34.7 cheater, yet *lasR* and *rhlR* expression remained unchanged, ruling out direct QS gene silencing. The genomic insertion, which includes genes such as an adenylate cyclase and a magnesium/cobalt transporter, could indirectly alter regulatory landscapes—perhaps by modulating cyclic AMP levels or the ion homeostasis impairing exoprotease production.

Alternatively, epigenetic silencing, such as DNA methylation or small RNA-mediated repression, might suppress exoprotease genes without altering QS regulators, a hypothesis supported by the transient non-producer phenotype in AUS 411. These possibilities warrant further investigation, as they could reveal novel layers of QS control in clinical isolates.

Environmental cues also modulate QS hierarchies, offering clues to AUS 531′s behavior. In PAO1, low phosphate inactivates *lasR*, elevating *rhlR* to the top of the QS cascade (Soto-Aceves et al. 2021). While our experiments used casein-rich conditions, CF lung environments—rich in amino acids, mucins, and stressors like oxidative bursts—may select for strains with flexible QS regulation. Early studies found that *lasR* transcription correlated with *lasB* and *aprA* expression in only half of clinical isolates (Cabrol et al. 2003), suggesting LasR-independent exoprotease control in some strains, although exoprotease activity was not assessed. Our proteomic data confirm that LasA and LasB are the dominant exoproteases in AUS 531. However, their loss in 34.7 without *lasR* or *rhlR* changes suggests additional regulatory inputs, possibly tied to metabolic adaptation or stress responses.

Metabolic differences further distinguish clinical isolates from reference strains. Some *lasR* mutants from CF isolates grow better in the presence of amino acids than their parental strains (D’Argenio et al. 2007), likely reflecting adaptation to peptide-rich sputum. In contrast, our AUS 531 *lasR* mutant grew slower in casamino acids than the wild-type. At the same time, the naturally evolved 34.7 cheater matched wild-type growth rates and outcompeted the *lasR* mutant in competitions. This fitness advantage may contribute to the lack of *lasR* mutant selection during evolution experiments, since their impaired assimilation of casein hydrolysis products likely offsets any cheating benefit. This metabolic cost may be strain-specific, as PAO1 *lasR* mutants showed no such growth defect, highlighting how clinical isolates integrate QS with metabolic networks differently from laboratory strains.

Another factor that may prevent the selection of AUS 531 *lasR* mutants in casein is their retained ability, to some extent, to grow using casein as the sole carbon source. Consequently, they can only mildly exploit the exoproteases of cooperators. Interestingly, AUS 531 *lasR* mutants can grow in the presence of casein, despite exhibiting low caseinolytic activity in their supernatants. This phenomenon requires further investigation to determine the mechanisms enabling growth. One possibility is that they degrade casein using membrane-associated proteases, such as ICMP (Fricke et al. 1999).

Moreover, non-social adaptations that improve casein metabolism in cooperators, such as those that inactivate the transcriptional repressor *psdR* confer protection against exoprotease exploitation and prevent the tragedy of the commons in PAO1 (Asfahl et al. 2015, Robinson et al. 2020), if this is also the case for the strains AUS 411 and AUS 531 studied here, it needs further investigation. The deviations from the reference strain exoprotease cheating paradigm identified here may have broader implications for *P. aeruginosa* sociobiology and pathogenesis. The mild cheating and stable growth of AUS 531 suggest some clinical isolates evolve cooperation networks resistant to exploitation, potentially enhancing persistence in chronic infections like CF. This resilience could reduce the efficacy of therapeutic strategies that introduce cheaters to outcompete cooperators, as tested in PAO1 and PA14 in infected wounds (Rumbaugh et al. 2009, Mutlu et al. 2023). If exoprotease production *in vivo* mirrors our findings—sustained despite cheating attempts—such approaches might fail against adapted strains. Especially if those strategies are attempted for chronic lung infections such as those present in CF patients, in which it is common that the infective strains are well adapted for persistence and have adaptive mutations in their QS circuitry (Winstanley et al. 2016). More investigation is needed to determine if these outcomes are less likely in other infections such as wounds, since to date a limited number of isolates (PAO1, PA14, and P729) from those niches are characterized regarding their cheating behavior (Diggle et al. 2007, Sandoz et al. 2007, Dandekar et al. 2012, Loarca et al. 2019).

Our results incentivize further studies across diverse *P. aeruginosa* isolates, both *in vitro* and *in vivo*, using infection models like murine airways or CF sputum mimics. Characterizing the mechanistic basis of exoprotease loss in AUS 531 34.7—whether genomic rearrangements, epigenetic silencing, or unidentified regulators—could uncover new QS regulatory pathways. Additionally, testing cheater-based therapies against isolates like AUS 531 in realistic infection contexts could refine their clinical potential, addressing whether robust QS networks limit their utility.

In conclusion, clinical *P. aeruginosa* isolates, such as AUS 531 and AUS 411, reveal a spectrum of social behaviors and QS regulation. These isolates exhibit a spectrum of social behaviors and QS regulation that is not predicted by reference strain paradigms. Their resistance to exoprotease cheating and population collapse suggests evolutionary adaptations that sustain cooperation, potentially enhancing virulence and persistence. Elucidating these mechanisms will deepen our understanding *of P. aeruginosa* sociobiology and inform targeted therapies for chronic infections, moving beyond the one-size-fits-all models derived from PAO1 and PA14 reference strains.

## Supplementary Material

fiaf106_Supplemental_Files
