# Supplementary material for: Challenging the paradigm: non-canonical exoprotease cheating in clinical Pseudomonas aeruginosa isolates
Source: FEMS Microbiol Ecol. 2025 Oct 22;101(11):fiaf106. doi: 10.1093/femsec/fiaf106 (PMC12574336; doi:10.1093/femsec/fiaf106)
Supplement: fiaf106_Supplemental_Files [file fiaf106_supplemental_files.zip › Supple fig non canonical 20-8-25.pptx]

## Slide 1
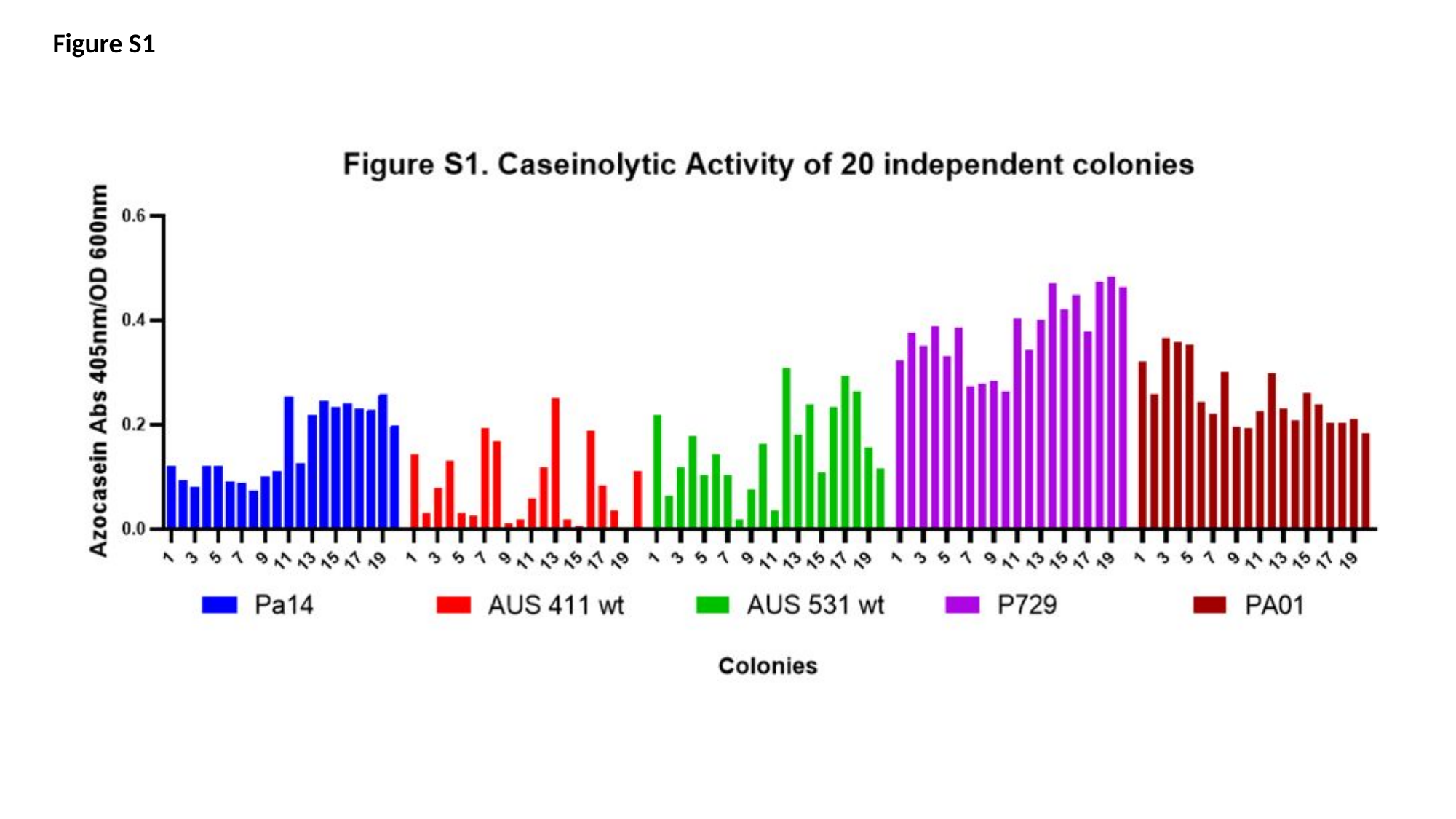

Figure S1

## Slide 2
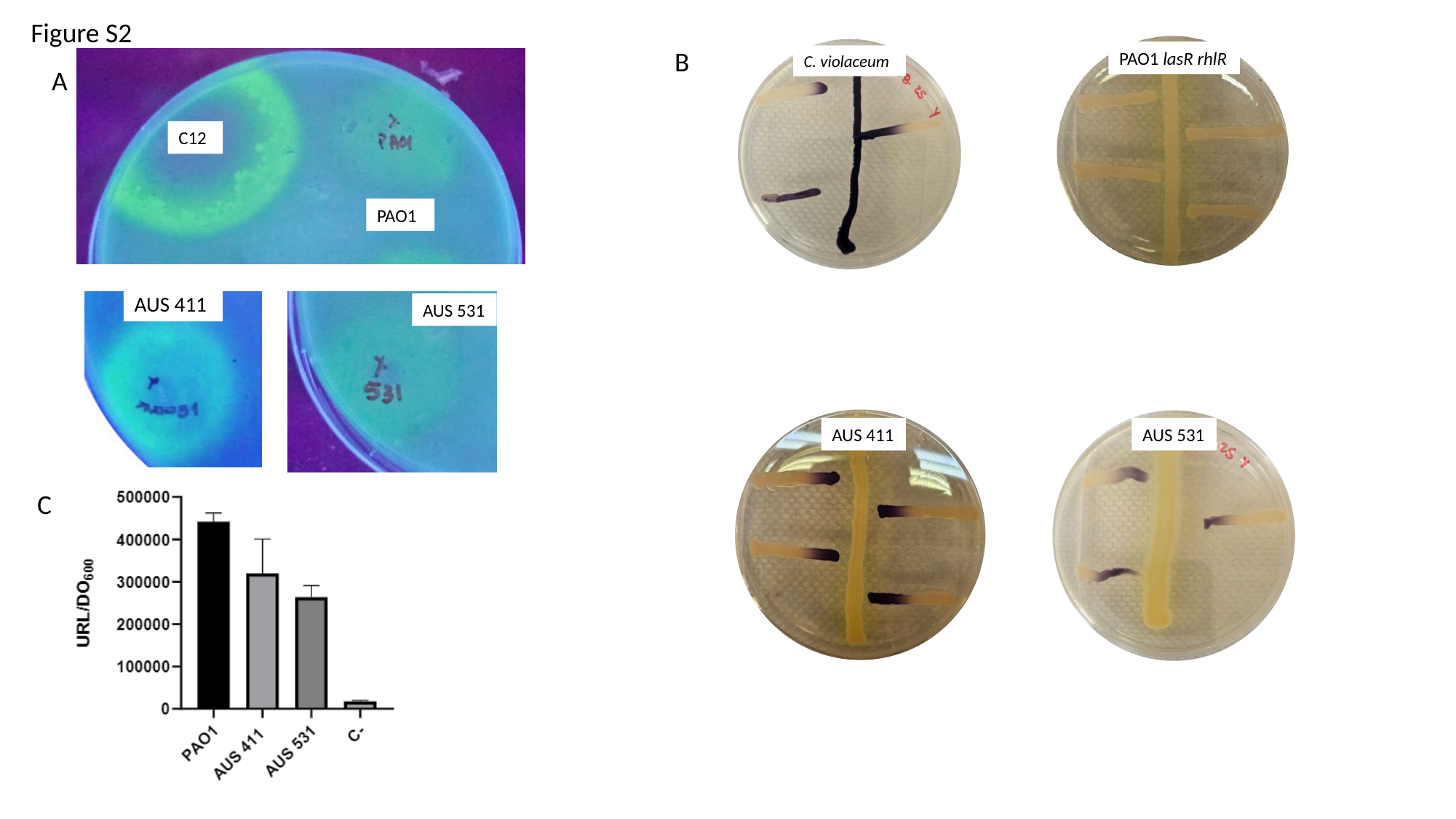

Figure S2
B
PAO1 lasR rhlR
C. violaceum
A
C12
PAO1
AUS 411
AUS 531
AUS 411
AUS 531
C

## Slide 3
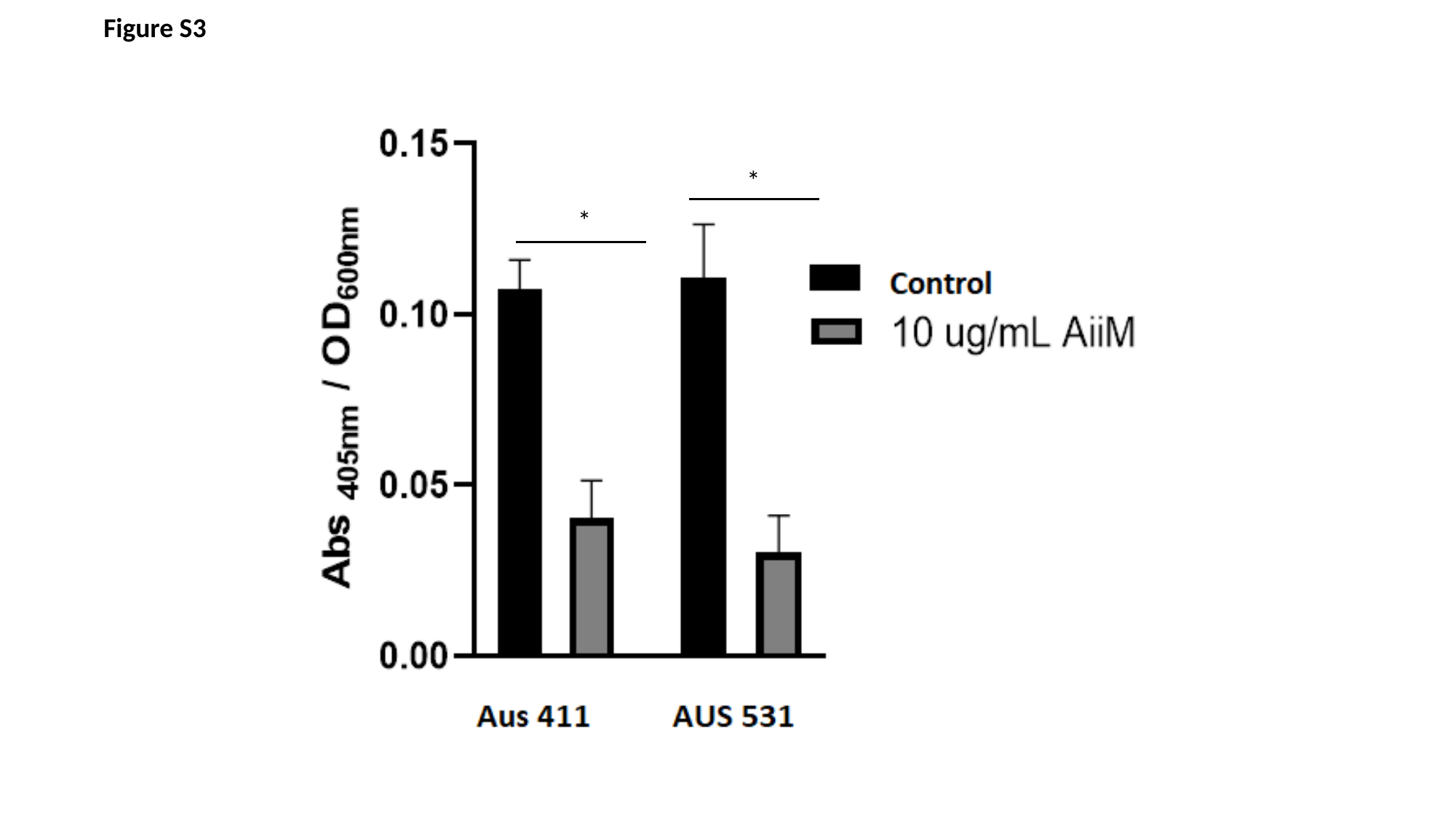

Figure S3
*
*

## Slide 4
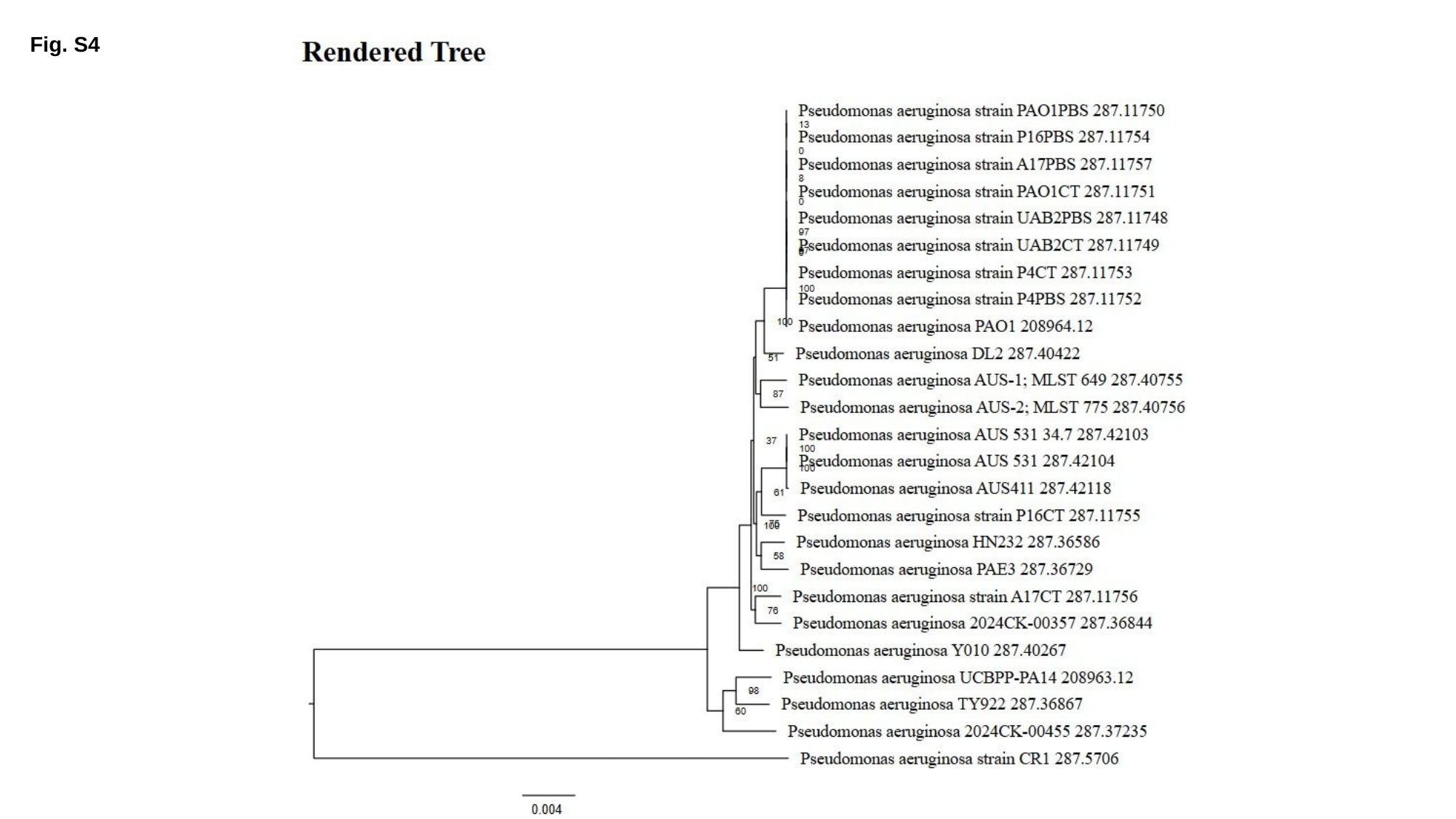

Fig. S4

## Slide 5
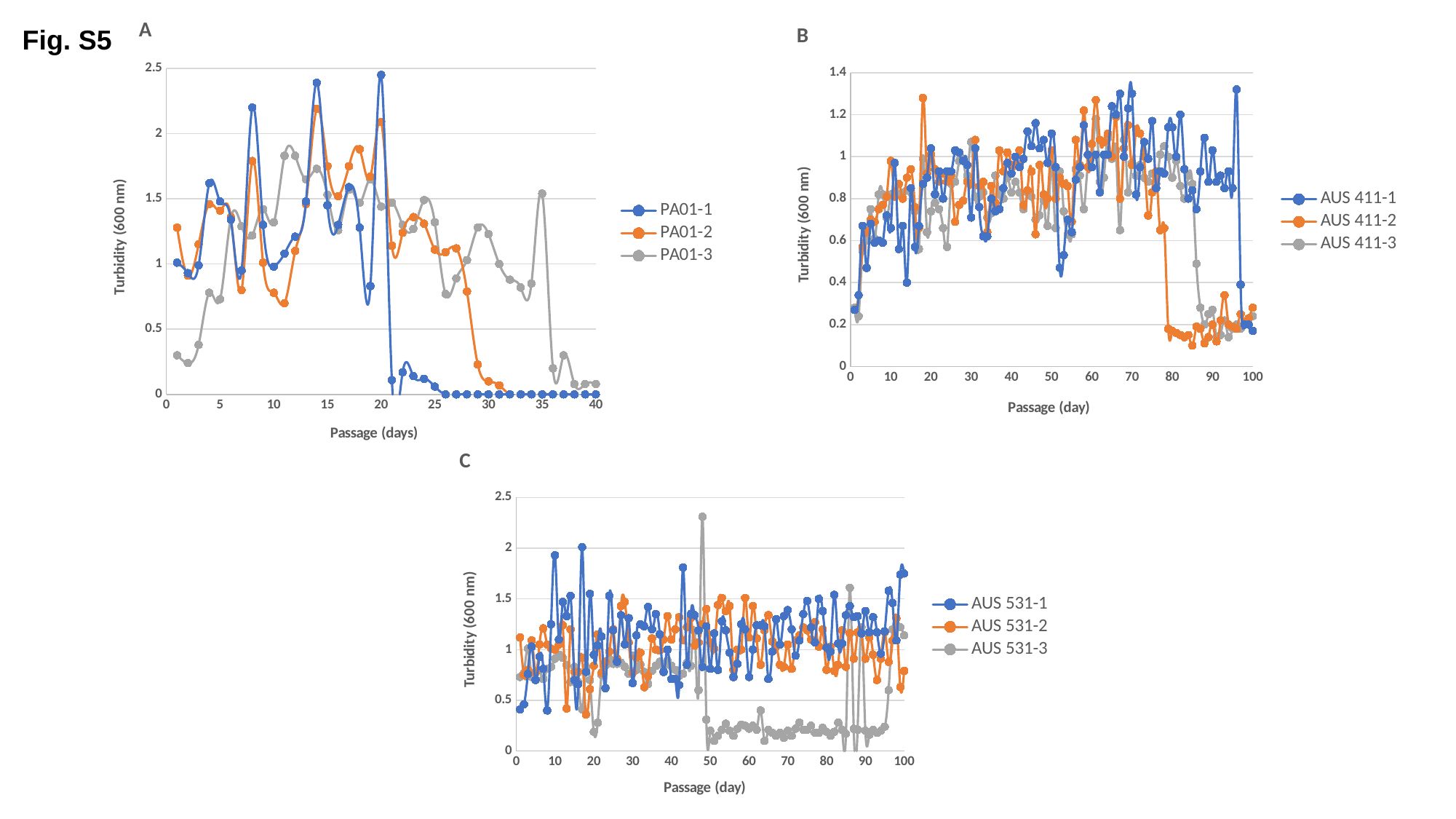

### Chart: A
| Category | PA01-1 | PA01-2 | PA01-3 |
|---|---|---|---|
### Chart: B
| Category | AUS 411-1 | AUS 411-2 | AUS 411-3 |
|---|---|---|---|Fig. S5
### Chart: C
| Category | AUS 531-1 | AUS 531-2 | AUS 531-3 |
|---|---|---|---|

## Slide 6
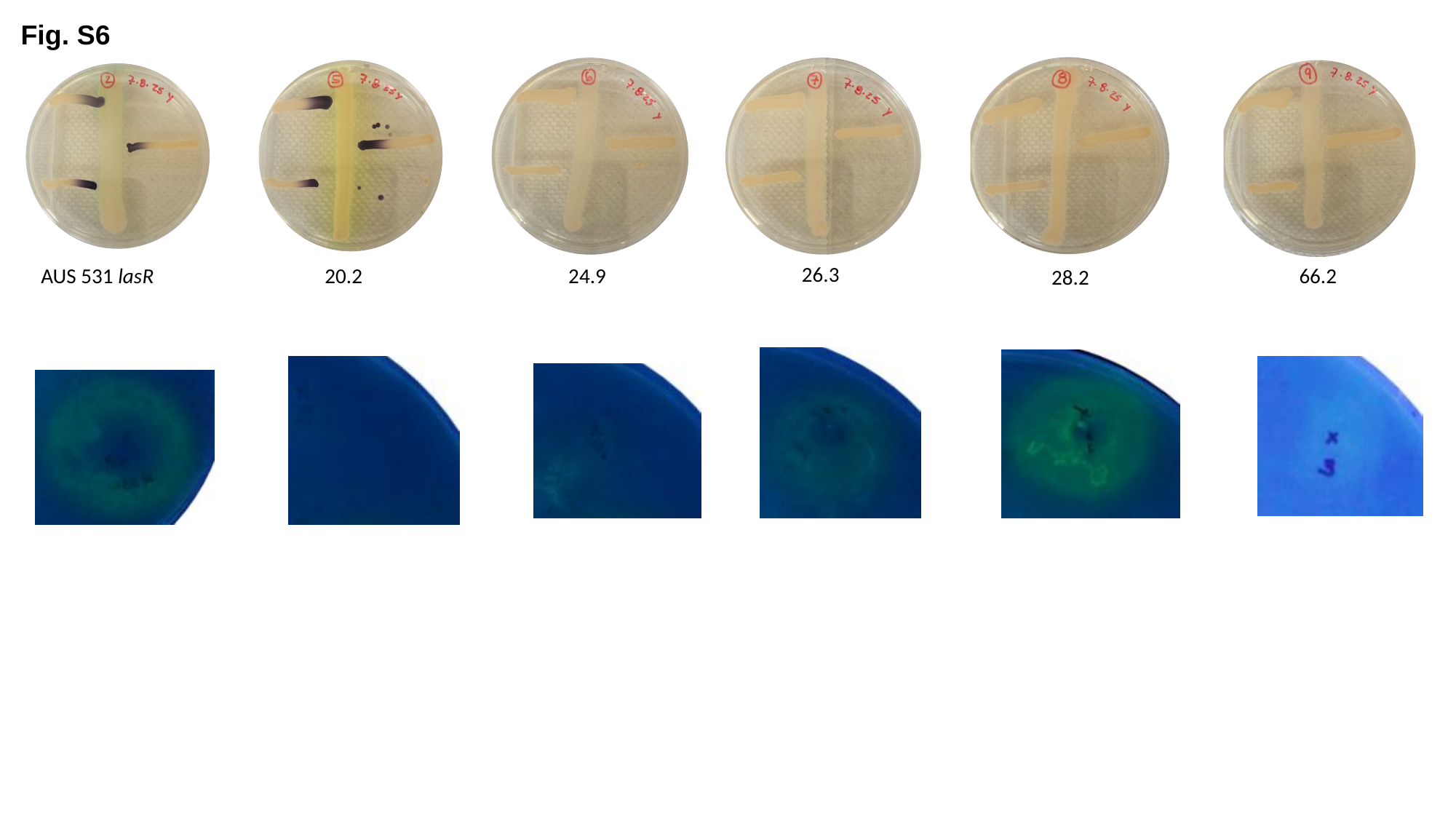

Fig. S6
26.3
66.2
20.2
24.9
AUS 531 lasR
28.2

## Slide 7
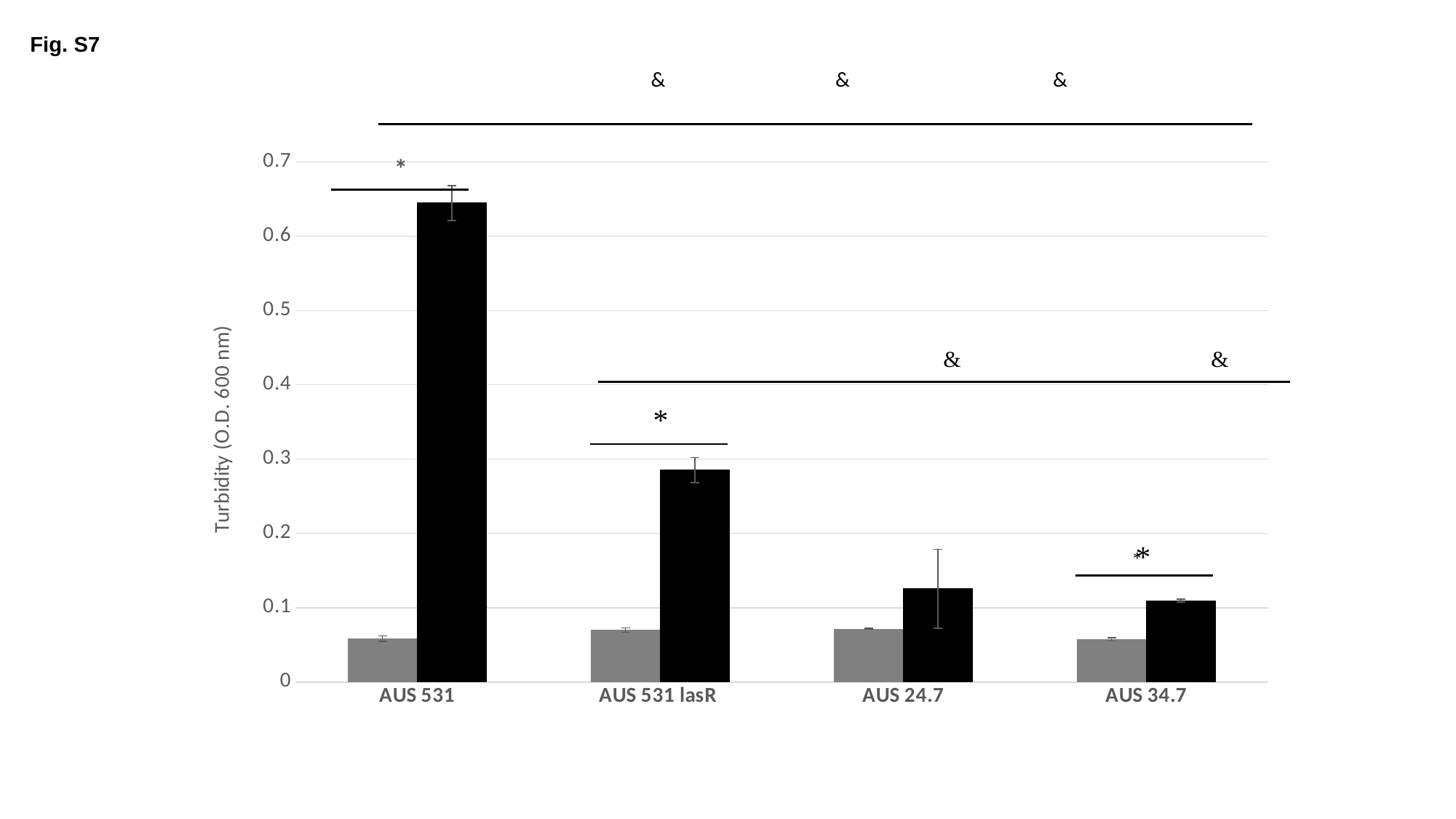

Fig. S7
 & & &
### Chart: *
| Category | 0 h | 18 h |
|---|---|---|
| AUS 531 | 0.058499999999999996 | 0.6445000000000001 |
| AUS 531 lasR | 0.06999999999999998 | 0.28500000000000003 |
| AUS 24.7 | 0.07149999999999998 | 0.1255 |
| AUS 34.7 | 0.057499999999999996 | 0.10949999999999999 |

## Slide 8
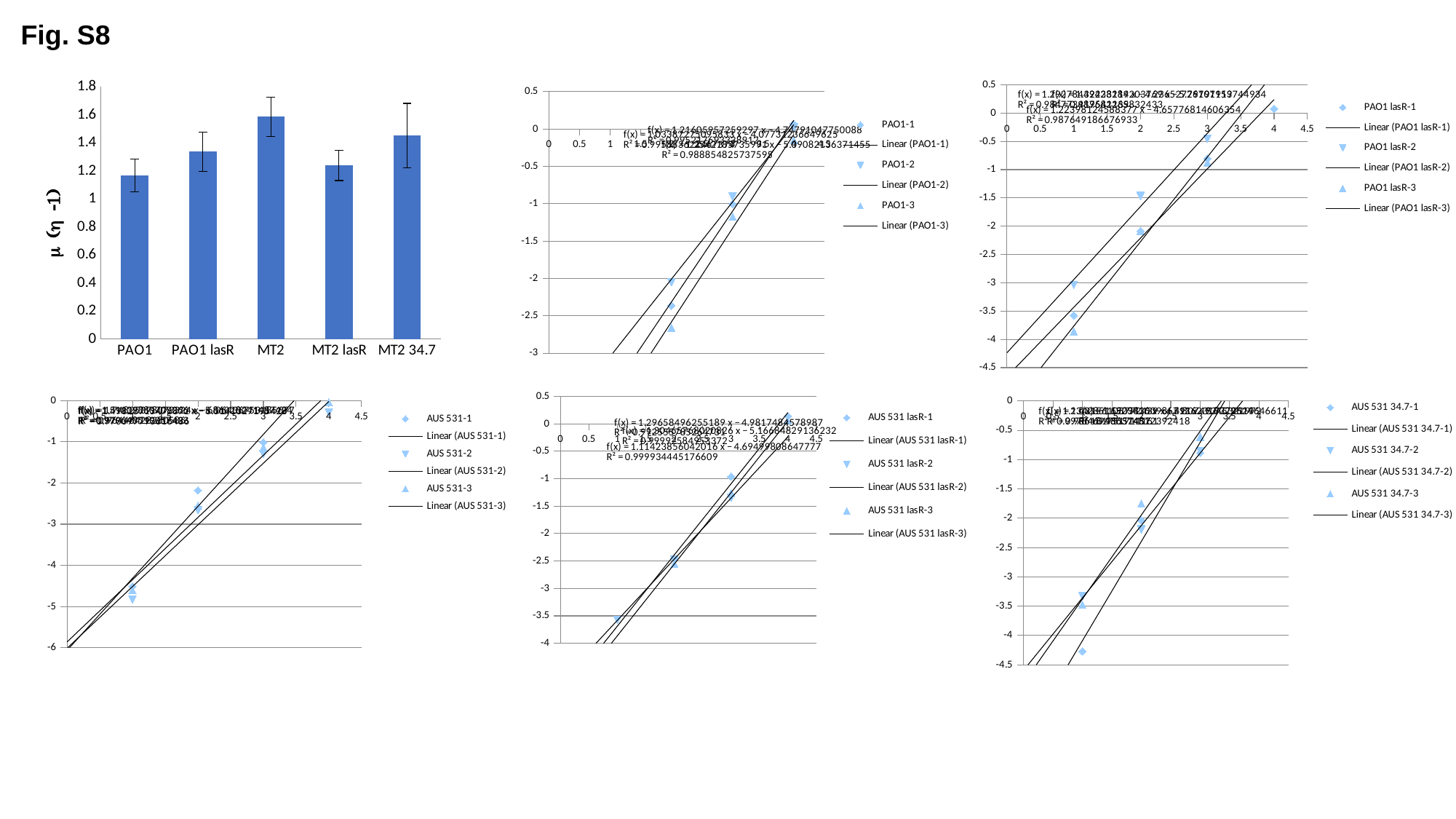

Fig. S8
### Chart
| Category | |
|---|---|
| PAO1 | 1.1680666666666666 |
| PAO1 lasR | 1.3370333333333333 |
| MT2 | 1.586 |
| MT2 lasR | 1.2385 |
| MT2 34.7 | 1.4523 |
### Chart
| Category | PAO1 lasR-1 | PAO1 lasR-2 | PAO1 lasR-3 |
|---|---|---|---|
### Chart
| Category | PAO1-1 | PAO1-2 | PAO1-3 |
|---|---|---|---|
### Chart
| Category | AUS 531 lasR-1 | AUS 531 lasR-2 | AUS 531 lasR-3 |
|---|---|---|---|
### Chart
| Category | AUS 531-1 | AUS 531-2 | AUS 531-3 |
|---|---|---|---|
### Chart
| Category | AUS 531 34.7-1 | AUS 531 34.7-2 | AUS 531 34.7-3 |
|---|---|---|---|

## Slide 9
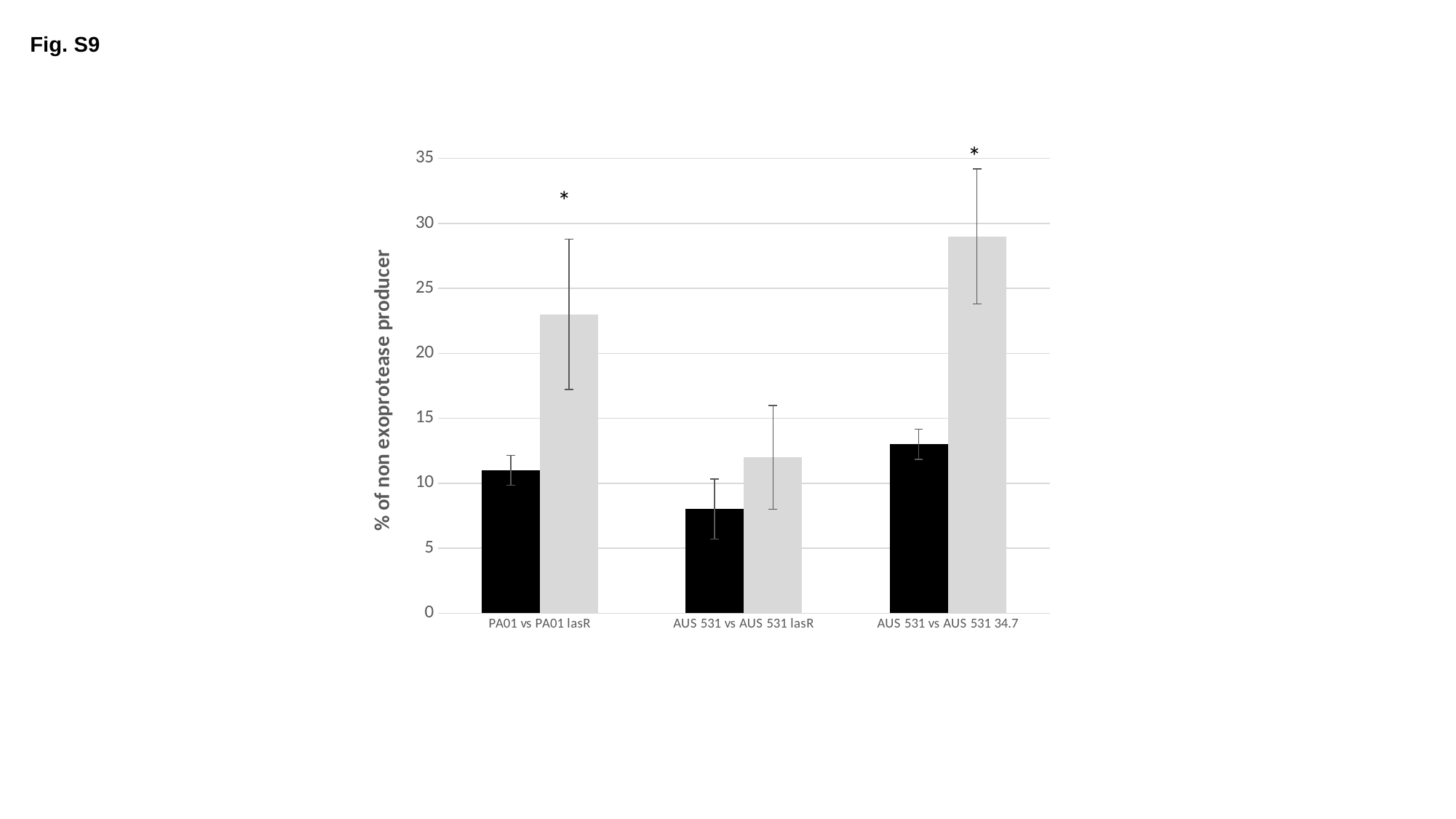

Fig. S9
*
### Chart
| Category | T0 | 24 h |
|---|---|---|
| PA01 vs PA01 lasR | 11.0 | 23.0 |
| AUS 531 vs AUS 531 lasR | 8.0 | 12.0 |
| AUS 531 vs AUS 531 34.7 | 13.0 | 29.0 |*

## Slide 10
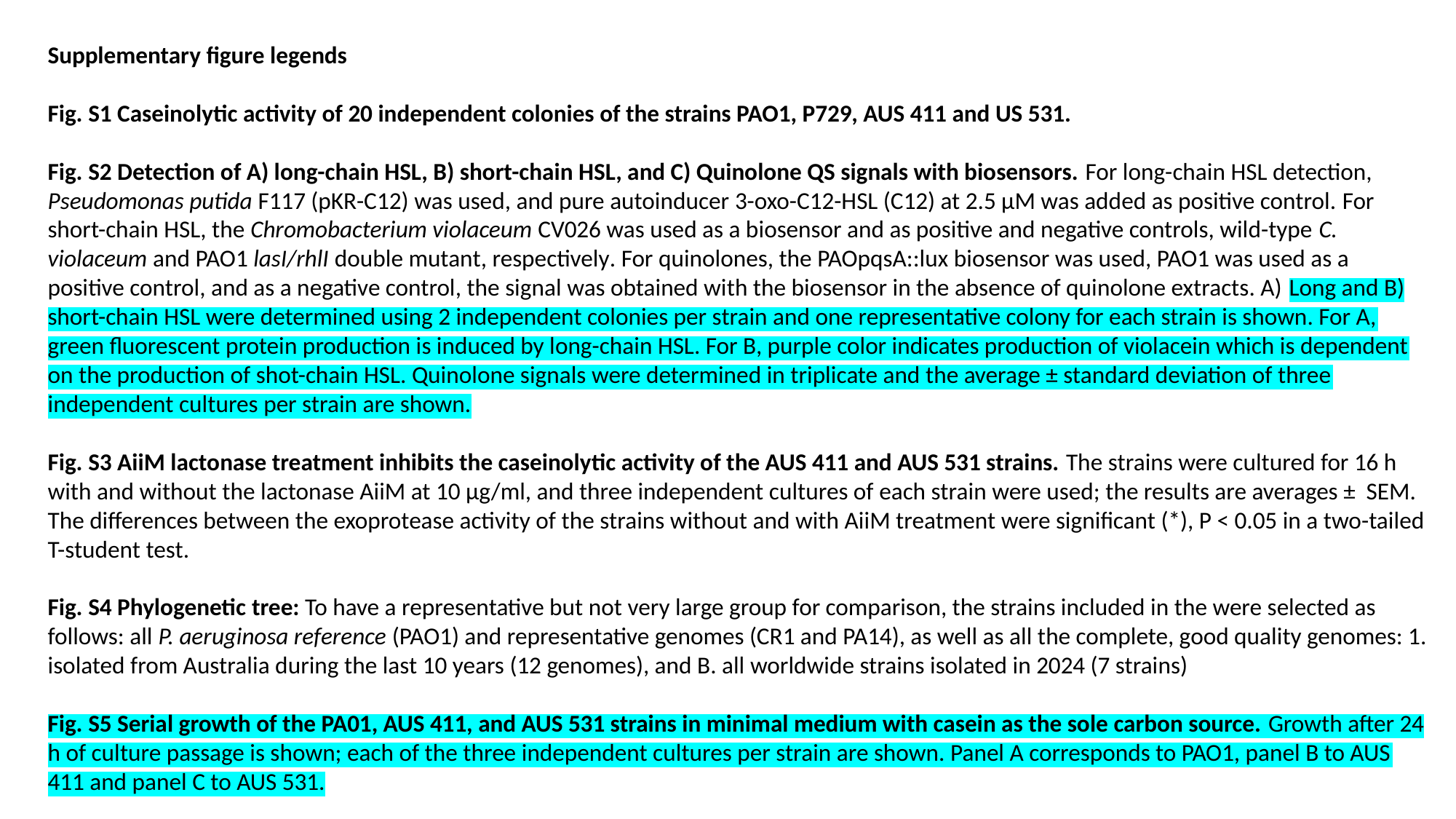

Supplementary figure legends
Fig. S1 Caseinolytic activity of 20 independent colonies of the strains PAO1, P729, AUS 411 and US 531.
Fig. S2 Detection of A) long-chain HSL, B) short-chain HSL, and C) Quinolone QS signals with biosensors. For long-chain HSL detection, Pseudomonas putida F117 (pKR-C12) was used, and pure autoinducer 3-oxo-C12-HSL (C12) at 2.5 µM was added as positive control. For short-chain HSL, the Chromobacterium violaceum CV026 was used as a biosensor and as positive and negative controls, wild-type C. violaceum and PAO1 lasI/rhlI double mutant, respectively. For quinolones, the PAOpqsA::lux biosensor was used, PAO1 was used as a positive control, and as a negative control, the signal was obtained with the biosensor in the absence of quinolone extracts. A) Long and B) short-chain HSL were determined using 2 independent colonies per strain and one representative colony for each strain is shown. For A, green fluorescent protein production is induced by long-chain HSL. For B, purple color indicates production of violacein which is dependent on the production of shot-chain HSL. Quinolone signals were determined in triplicate and the average ± standard deviation of three independent cultures per strain are shown.
Fig. S3 AiiM lactonase treatment inhibits the caseinolytic activity of the AUS 411 and AUS 531 strains. The strains were cultured for 16 h with and without the lactonase AiiM at 10 µg/ml, and three independent cultures of each strain were used; the results are averages ± SEM. The differences between the exoprotease activity of the strains without and with AiiM treatment were significant (*), P ˂ 0.05 in a two-tailed T-student test.
Fig. S4 Phylogenetic tree: To have a representative but not very large group for comparison, the strains included in the were selected as follows: all P. aeruginosa reference (PAO1) and representative genomes (CR1 and PA14), as well as all the complete, good quality genomes: 1. isolated from Australia during the last 10 years (12 genomes), and B. all worldwide strains isolated in 2024 (7 strains)
Fig. S5 Serial growth of the PA01, AUS 411, and AUS 531 strains in minimal medium with casein as the sole carbon source. Growth after 24 h of culture passage is shown; each of the three independent cultures per strain are shown. Panel A corresponds to PAO1, panel B to AUS 411 and panel C to AUS 531.

## Slide 11
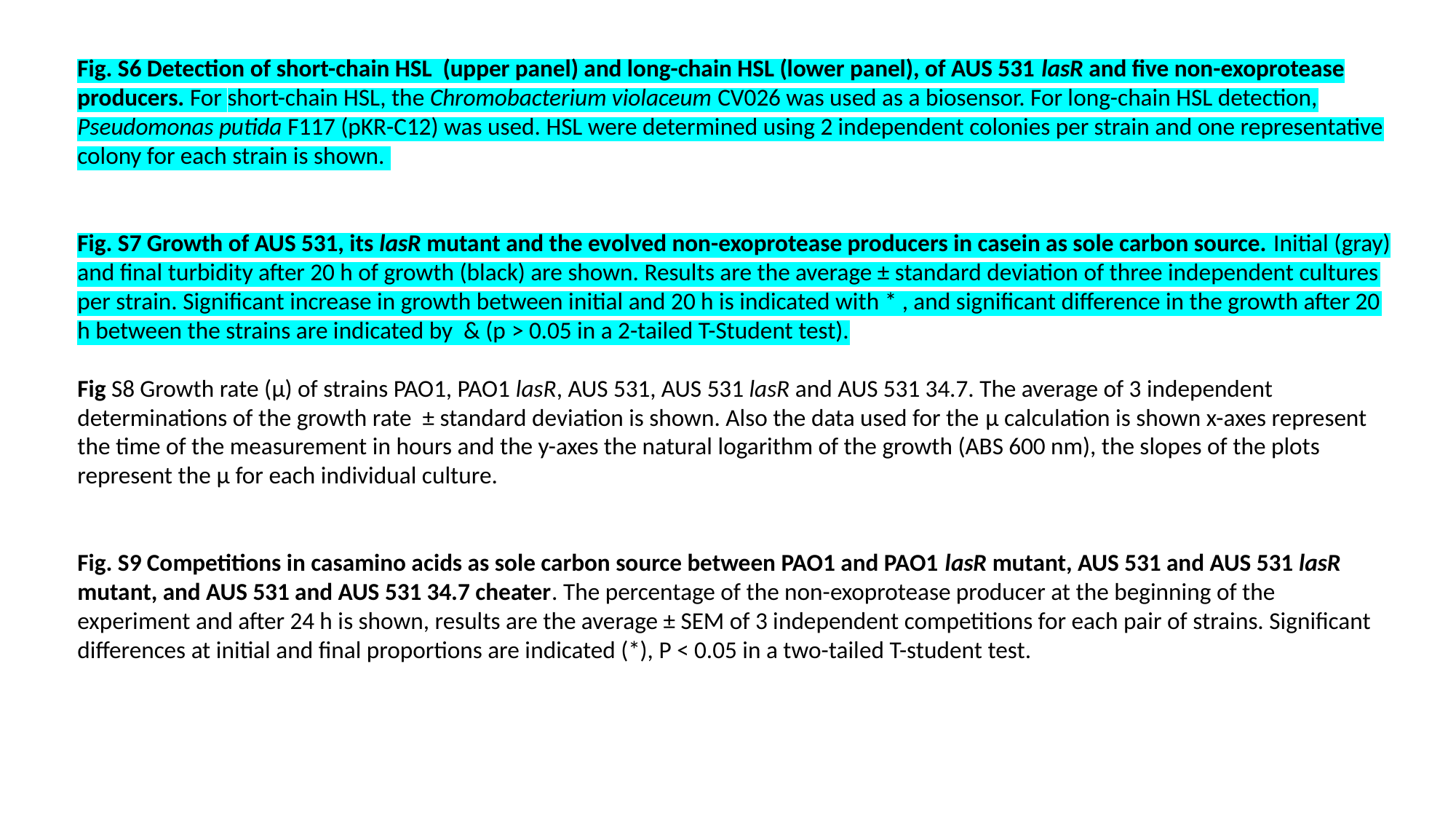

Fig. S6 Detection of short-chain HSL (upper panel) and long-chain HSL (lower panel), of AUS 531 lasR and five non-exoprotease producers. For short-chain HSL, the Chromobacterium violaceum CV026 was used as a biosensor. For long-chain HSL detection, Pseudomonas putida F117 (pKR-C12) was used. HSL were determined using 2 independent colonies per strain and one representative colony for each strain is shown.
Fig. S7 Growth of AUS 531, its lasR mutant and the evolved non-exoprotease producers in casein as sole carbon source. Initial (gray) and final turbidity after 20 h of growth (black) are shown. Results are the average ± standard deviation of three independent cultures per strain. Significant increase in growth between initial and 20 h is indicated with * , and significant difference in the growth after 20 h between the strains are indicated by & (p ˃ 0.05 in a 2-tailed T-Student test).
Fig S8 Growth rate (μ) of strains PAO1, PAO1 lasR, AUS 531, AUS 531 lasR and AUS 531 34.7. The average of 3 independent determinations of the growth rate ± standard deviation is shown. Also the data used for the μ calculation is shown x-axes represent the time of the measurement in hours and the y-axes the natural logarithm of the growth (ABS 600 nm), the slopes of the plots represent the μ for each individual culture.
Fig. S9 Competitions in casamino acids as sole carbon source between PAO1 and PAO1 lasR mutant, AUS 531 and AUS 531 lasR mutant, and AUS 531 and AUS 531 34.7 cheater. The percentage of the non-exoprotease producer at the beginning of the experiment and after 24 h is shown, results are the average ± SEM of 3 independent competitions for each pair of strains. Significant differences at initial and final proportions are indicated (*), P ˂ 0.05 in a two-tailed T-student test.
