# Supplementary material for: Challenging the paradigm: non-canonical exoprotease cheating in clinical Pseudomonas aeruginosa isolates
Source: FEMS Microbiol Ecol. 2025 Oct 22;101(11):fiaf106. doi: 10.1093/femsec/fiaf106 (PMC12574336; doi:10.1093/femsec/fiaf106)
Supplement: fiaf106_Supplemental_Files [file fiaf106_supplemental_files.zip › Table S4.docx]

**Table S4.** ***Pseudomonas aeruginosa*** **strains used in this work**

| **Strain** | **Characteristics** | **Source** |
| --- | --- | --- |
| PAO1 | Reference strain, representative of clade 1 | (Soto-Aceves *et al.*, 2021) |
| PAO1 *lasR* | *lasR* gene interrupted with apramycin cassette | (Soto-Aceves *et al.*, 2021) |
| AUS 411 | Clinical isolate/ international CF clone ST274-CC274 | (Ambroa *et al.*, 2020) |
| AUS 531 | Clinical isolate/ international CF clone ST274-CC274 | (Ambroa *et al.*, 2020) |
| AUS 531 *lasR* | *lasR* gene interrupted with apramycin cassette | this work |
| AUS 531 24.7 | Evolved non exoprotease producer | this work |
| AUS 531 34.7 | Evolved non exoprotease producer | this work |
| AUS 531 20.2 | Evolved non exoprotease producer | this work |
| AUS 531 24.9 | Evolved non exoprotease producer | this work |
| AUS 531 26.3 | Evolved non exoprotease producer | this work |
| AUS 531 28.2 | Evolved non exoprotease producer | this work |
| AUS 531 66.2 | Evolved non exoprotease producer | this work |
